# Functionality of the Crosswise Model for Assessing Sensitive or Transgressive Behavior: A Systematic Review and Meta-Analysis

**DOI:** 10.3389/fpsyg.2021.655592

**Published:** 2021-06-23

**Authors:** Dominic Sagoe, Maarten Cruyff, Owen Spendiff, Razieh Chegeni, Olivier de Hon, Martial Saugy, Peter G. M. van der Heijden, Andrea Petróczi

**Affiliations:** ^1^Department of Psychosocial Science, University of Bergen, Bergen, Norway; ^2^Faculty of Social Sciences, Utrecht University, Utrecht, Netherlands; ^3^School of Life Sciences, Pharmacy and Chemistry, Kingston University, London, United Kingdom; ^4^Doping Authority Netherlands, Capelle aan den IJssel, Netherlands; ^5^Institute of Sport Sciences, University of Lausanne, Lausanne, Switzerland; ^6^Statistical Science Southampton Research Institute, University of Southampton, Southampton, United Kingdom; ^7^Department of Movement Sciences, KU Leuven, Leuven, Belgium

**Keywords:** randomized response, crosswise model, direct question, prevalence, quality assessment, efficiency, survey

## Abstract

Tools for reliable assessment of socially sensitive or transgressive behavior warrant constant development. Among them, the Crosswise Model (CM) has gained considerable attention. We systematically reviewed and meta-analyzed empirical applications of CM and addressed a gap for quality assessment of indirect estimation models. Guided by the PRISMA protocol, we identified 45 empirical studies from electronic database and reference searches. Thirty of these were comparative validation studies (CVS) comparing CM and direct question (DQ) estimates. Six prevalence studies exclusively used CM. One was a qualitative study. Behavior investigated were substance use and misuse (*k* = 13), academic misconduct (*k* = 8), and corruption, tax evasion, and theft (*k* = 7) among others. Majority of studies (*k* = 39) applied the “more is better” hypothesis. Thirty-five studies relied on birthday distribution and 22 of these used *P* = 0.25 for the non-sensitive item. Overall, 11 studies were assessed as high-, 31 as moderate-, and two as low quality (excluding the qualitative study). The effect of non-compliance was assessed in eight studies. From mixed CVS results, the meta-analysis indicates that CM outperforms DQ on the “more is better” validation criterion, and increasingly so with higher behavior sensitivity. However, little difference was observed between DQ and CM estimates for items with DQ prevalence estimate around 50%. Based on empirical evidence available to date, our study provides support for the superiority of CM to DQ in assessing sensitive/transgressive behavior. Despite some limitations, CM is a valuable and promising tool for population level investigation.

## Introduction

Social desirability bias has been identified as emanating from: (1) fear of exposure and consequences, and/or (2) self-presentation concern (Tourangeau and Yan, 2007; Krumpal, 2013). Indirect estimation models (IEM) using randomization (randomized response models: RRM) or a fuzzy response mode (fuzzy response models: FRM) aim to address fear of exposure and consequences by offering protection beyond anonymity (Lensvelt-Mulders et al., 2005a). Due to the format of IEM, researchers cannot relate responses to the sensitive item (question or statement) to individual respondents. Several models have been developed (Lensvelt-Mulders et al., 2005a; Nuno and St. John, 2015; Chaudhuri, 2016; Pitsch, 2016; Rao and Rao, 2016) characterized by the deliberate inclusion of “statistical noise” for respondents' protection. Thus, whilst researchers cannot find out how individuals respond to a sensitive item in IEM, *a priori* knowledge of the probability distribution of the “statistical noise” allows researchers to estimate the proportion of affirmative answers to the sensitive item.

RRM typically employ a device (e.g., dice, pack of cards) or a method (e.g., number distributions such as birthdays) to direct participants to which item to respond to; or administer two items (the sensitive target item paired with a non-sensitive or innocuous item). Examples of RRM include the Randomized Response Technique (Dalton and Metzger, 1992), the Warner method or mirrored questions (Warner, 1965), the Unrelated Question Model (Greenberg et al., 1969), and Forced Responses (Boruch, 1971). In contrast to RRM, instead of relying on randomization for the items, FRM add uncertainty to the response options by making the response “vague.” Examples of FRM include the Unmatched List (Droitcour et al., 1991), Single Sample Count (Petróczi et al., 2011; Nepusz et al., 2014), and the Crosswise Model (CM: Yu et al., 2008).

**Figure 1:**
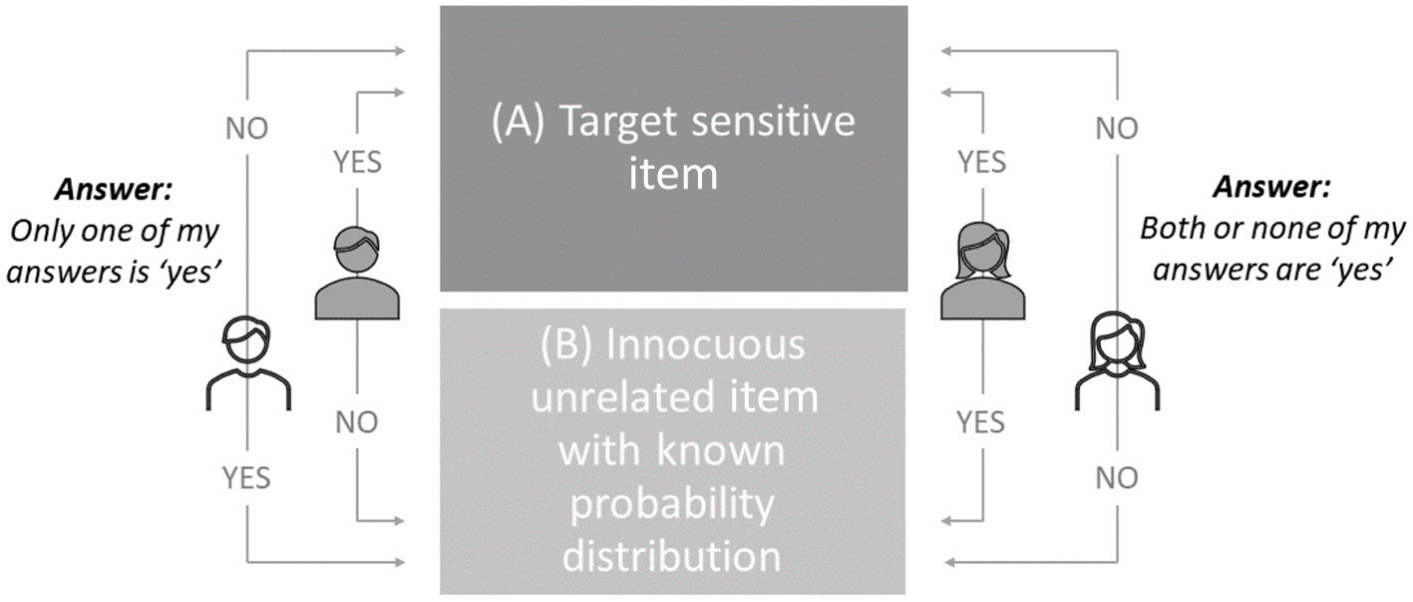
Conceptual framework of CM.

In using CM, participants are presented with a sensitive target item paired with an innocuous item. Participants are then presented with two response options: one “yes” answer (or “true” statement) without revealing which one, or either none or two “yes” answers (or “true” statements) without revealing if it is none or both, with the innocuous item having a known probability of an affirmative response (e.g., *P* = 0.25 means that 25% of the respondents are expected to give an affirmative answer). As the response options are deliberately fuzzy, it is impossible to find out how the person responded to the sensitive item. As depicted in Figure 1, the same response option can equally include an affirmative or negative answer to the sensitive item.

CM has gained popularity over other IEM due to its advantages of simplicity (simple instructions, one-step process), suitability for self-administration (no need for a randomization device), and the absence of a forced answer. Furthermore, as both response options contain a possible affirmative answer to the sensitive item, there is no obvious self-protection strategy by favoring one response option to avoid suspicion. The aims of this study were to systematically review and meta-analyze evidence on applications of CM in empirical research, as well as assess its performance. For the latter aim, we developed and applied a quality assessment criteria. For the meta-analysis, we hypothesized that for items measuring sensitive or transgressive behavior, CM yields a higher prevalence estimate than direct questioning.

## Methods

### Search Strategy and Inclusion Criteria

We conducted a systematic literature search in PubMed, ScienceDirect, and Scopus. The following keywords were used: “crosswise model,” “crosswise AND prevalence,” “crosswise AND model AND prevalence,” and “crosswise AND estimat^*^”. The key inclusion criteria were that the study presented: (a) empirical or original research, (b) assessing sensitive or transgressive behavior or attribute, (c) using CM or its variant, and (d) is a full scientific article (not a book chapter, conference abstract, or editorial) published in English. *Ad hoc* searches were also conducted as part of our comprehensiveness assurance process. The latest literature search was conducted on 17th March, 2021. We conducted the literature search and selection in line with the Preferred Reporting Items for Systematic Reviews and Meta-Analyses (PRISMA) procedure (Moher et al., 2009).

### Data Extraction and Synthesis

The first and last authors (DS, AP) conducted the literature search and selection of articles based on the aforementioned criteria. Using a standardized data extraction form, the following data were extracted from the identified studies: first author name and publication year, sensitive behavior, focus of our review, behavior item, innocuous item, independency of innocuous items, sample type, sample size, study measure/instrument, method for split sample, study hypothesis, prevalence of sensitive behavior (%), and prevalence difference (Δ), and/or 95% Confidence Interval [95% CI], and/or standard error (±*SE*). See Supplementary Tables 1, 2. The first author (DS) conducted the data extraction, study analysis and synthesis using content analysis (Finfgeld-Connett, 2014).

### Quality Assessment

The quality of included studies was assessed using a twenty-item instrument (Supplementary Table 3) that combines 10 criteria for the assessment of the quality or risk of bias of prevalence studies (Hoy et al., 2012) with 10 criteria for the assessment of the quality or risk of bias of studies using IEM. The 10 items for the assessment of IEM were collated and evaluated by the researchers in the group with experience and expertise in IEM (AP, MC, PvdH, and OdH).

The lead author (DS) independently assessed the quality of included studies. Additionally, four reviewers (DS, OS, RC, AP) assessed the quality of included studies as a group. As study quality is signified by the absence of “penalty points,” lower overall scores indicate higher quality or lower risk of bias. For accessibility and comparability, we adopted the Grading of Recommendations Assessment, Development and Evaluation (GRADE) approach (Guyatt et al., 2008) which yields a quality assessment on one of four grades: high quality, moderate quality, low quality, and very low quality. Here, included studies were categorized as: high quality/low risk of bias (<25%), moderate quality/risk of bias (25–50%), low quality/high risk of bias (51–75%), and very low quality/very high risk of bias (>75%). These cut-off points reflect the absolute quartiles where the minimum score of zero represents the highest quality and total lack of bias, and a score of 20 (CM prevalence studies) or 10 (CM testing studies) is the lowest possible quality (see Supplementary Table 4).

### Meta-Analysis

We conducted a meta-analysis to compare CM prevalence estimates to those from direct question(s) (DQ). Based on the observed CM parameters in various applications, we calculated the standard error (*SE*) as a function of the probability of the sensitive behavior and probability of the affirmative response to the innocuous item for various sample sizes. In comparative validation studies (CVS: Höglinger and Jann, 2018), participants respond to the same sensitive item under DQ and CM, and effectivity is investigated by examining the difference between prevalence estimates from DQ and CM. In the present meta-analysis, we applied the same approach using multilevel analysis for the subset of studies where DQ was applied alongside CM. The effect of condition (CM vs. DQ) is computed as the difference in prevalence estimates on the probit scale. The difference score *d* is computed as:

$$\begin{array}{l} d_{probit}^{\prime }={Ẑ}_{CM}-{Ẑ}_{DQ} \end{array}$$

with $Ẑ=\Phi^{-1}\left(\hat{\pi }\right)$, where $\hat{\pi }$ is the prevalence estimate of the model, and Φ^−1^(·) is the inverse of the cumulative distribution function of the standard normal. Thus, the *d* score expresses the difference between the prevalence estimates in *z* scores, with positive scores denoting a higher CM prevalence estimate. For items measuring a socially desirable attribute, we relied on the negative of the *d* score. The data contained three items (Roberts and St. John, 2014; Shamsipour et al., 2014; Höglinger and Diekmann, 2017) with DQ prevalence estimates of 0, and one item (Safiri et al., 2019) with a CM estimate of 0 (yielding an infinite *z* score). In order not to discard these items from our analysis, and considering it is not unrealistic to assume that the prevalence in the population is not exactly 0, we set the *z* score for these items to −3.5 which is a little below the z score of −3.1 for the items with a DQ and CM prevalence estimate of 1%. Additionally, four items with negative CM prevalence estimates (Roberts and St. John, 2014; Jerke et al., 2021) were truncated at 0.

To account for the nesting of items within studies, we performed a multilevel analysis on the difference scores. To examine the dependence of the *d* score on the sensitivity of the item, we calculated a proxy for sensitivity as the absolute value of *Z*_*DQ*_. This score is 0 if the prevalence estimate in the DQ condition is 50% and increases as the estimate approximates to 0 or 1. The rationale for using this proxy is that, in general, the presence of attributes with low prevalence as well as the absence of attributes with high prevalence is perceived as deviations from the norm and therefore more sensitive. Although there may be exceptions to this general rule, it is advantageous that sensitivity is objectively assessed using the prevalence estimates of the items. Panel ratings of item sensitivity (Lensvelt-Mulders et al., 2005a) is a more subjective alternative. Details of the studies included in the meta-analysis are presented in Supplementary Table 5. The meta-analysis was conducted using R version 4.0.5 (R Core Team, 2021) with the lme4 (Bates et al., 2015) and tidyverse (Wickham et al., 2019) packages.

### Authors' Collaboration

Collaboration between authors as well as multiple publications by the same research group were notable in the eligible studies. We therefore conducted further scientometric analysis based on author names and publication year. Authorship network map and basic network properties were generated using Cytoscape version 3.8.2 (Shannon et al., 2003) with NetworkAnalyzer plug-in.

## Results

### Study Selection

**Figure 2:**
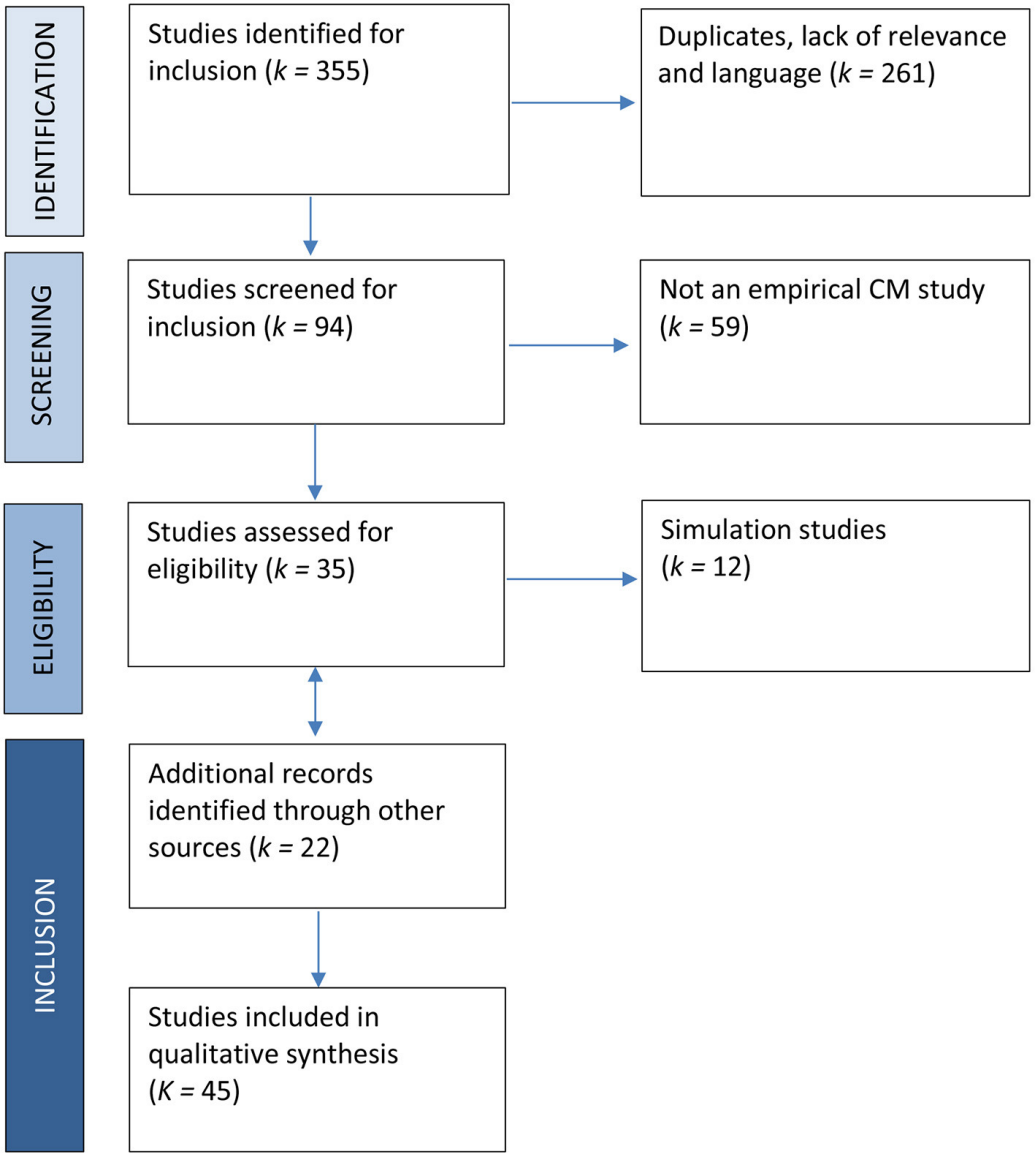
Flow diagram of systematic literature search on empirical applications of CM to assess sensitive/transgressive behavior.

A total of 355 hits were identified from the database search, and 261 were excluded for duplication, lack of relevance, or language. After screening the remaining 94 records, 59 records that are not empirical CM studies were excluded after further evaluation. Of the remaining 35 records assessed for eligibility, 12 simulation studies were excluded for lack of empirical data. Additionally, 22 records were identified through *ad hoc* searches, including 10 papers from the updated search. Thus, 45 full-text records were included in the meta-synthesis. Figure 2 presents results of the literature search and selection process.

### Publication Years and Origin

**Figure 3:**
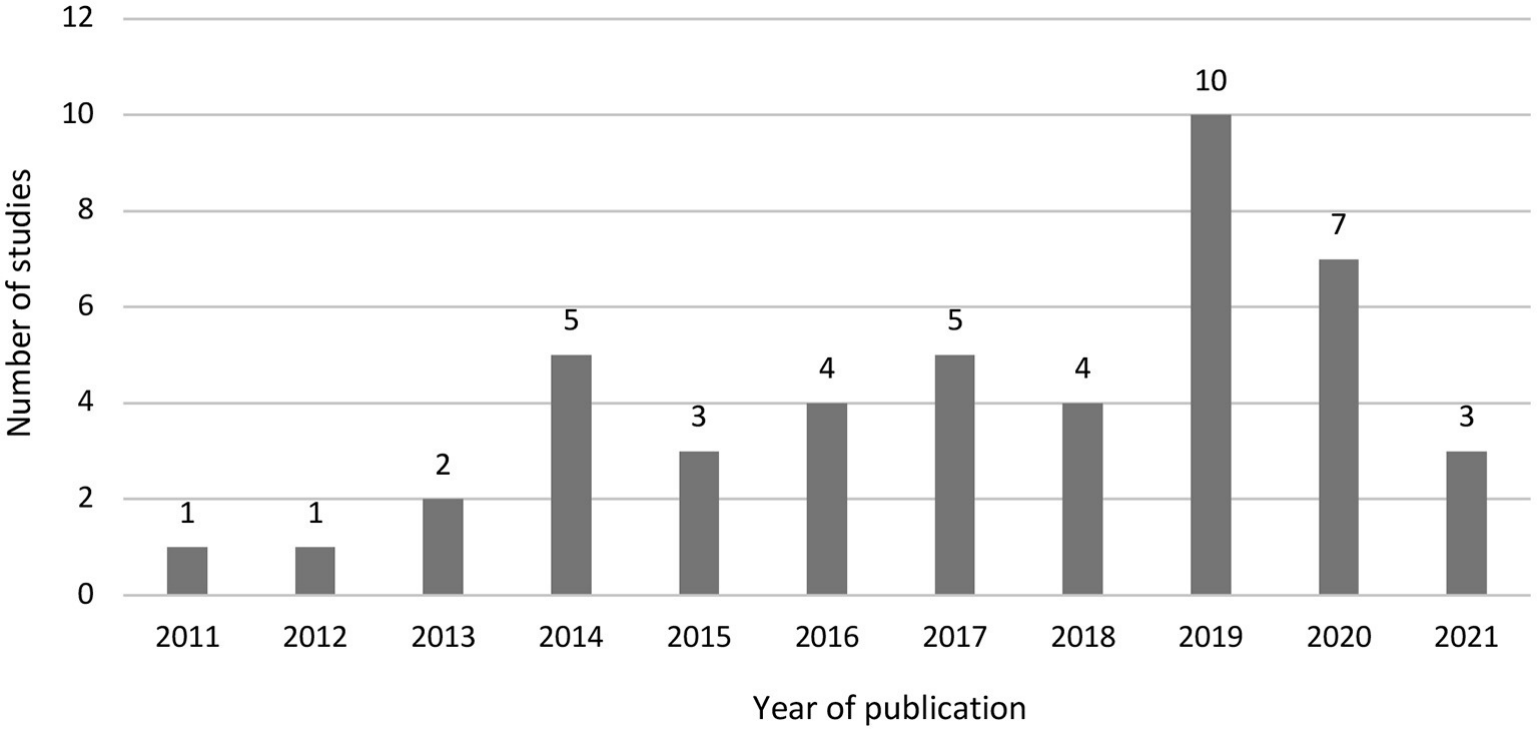
Number of CM studies by year.

Of the 45 included studies, publication years range from 2011 (Coutts et al., 2011) to 2021 (Canan et al., 2021; Jerke et al., 2021; Mieth et al., 2021). After a 3-year hiatus, on average 4–6 papers have been published each year (Figure 3). Studies originated from Germany (*k* = 16), Iran (*k* = 12), the US (*k* = 4), Switzerland (*k* = 3), Austria (*k* = 2), Costa Rica (*k* = 2), and one study each from Serbia, Turkey, and the UK. There were three international studies with samples from Germany and Switzerland (Jann et al., 2012), Germany, Switzerland and the UK (Jerke et al., 2019), and Austria, Germany, and Switzerland (Jerke et al., 2021).

### Study Type

In line with previous randomized response technique(s) (RRT) reviews (Umesh and Peterson, 1991; Lensvelt-Mulders et al., 2005a) and recent categorization (Höglinger and Jann, 2018), we classify CM applications for estimating the prevalence of sensitive or transgressive behavior as comparative, aggregate-level, and individual-level validation studies. We found 30 CVS that compared CM and DQ prevalence estimates. There were also nine aggregate-level validation studies that compared CM and DQ prevalence estimates to the true prevalence at the aggregate level, and one individual-level validation study that compared CM and DQ prevalence estimates to the true prevalence at the individual level. In addition to the above categorization, we identified six prevalence studies exclusively based on CM. See Table 1 for an overview of the studies, and Supplementary Tables 1, 2 for details of the studies. Three studies (Hoffmann et al., 2017; Jerke et al., 2019; Schnapp, 2019) were not included in the above categorization as they provided no CM prevalence estimates of sensitive or transgressive behavior.

**Table 1:** Summary of CM study type, sensitive/transgressive behavior investigated and results.

**Study type**	**Behavior**	**Result**	
		**CM% > DQ%**	**CM% < DQ%**
Comparative validation studies (*k* = 30)	Substance use and misuse	Nakhaee et al., 2013; Shamsipour et al., 2014; Höglinger et al., 2016; Mirzazadeh et al., 2018; Banayejeddi et al., 2019; Safiri et al., 2019; Özgül, 2020; Canan et al., 2021	Shamsipour et al., 2014; Mirzazadeh et al., 2018; Safiri et al., 2019
	Academic misconduct	Coutts et al., 2011; Jann et al., 2012; Roberts and St. John, 2014; Höglinger et al., 2016; Hopp and Speil, 2019; Jerke et al., 2021	Coutts et al., 2011; Jann et al., 2012; Roberts and St. John, 2014; Jerke et al., 2021
	Corruption, tax evasion, and theft	Korndörfer et al., 2014; Kundt, 2014; Gingerich et al., 2015; Höglinger and Jann, 2018; Hopp and Speil, 2019; Oliveros and Gingerich, 2020	
	Health and STDs	Mirzazadeh et al., 2018; Nasirian et al., 2018	
	Sexual behavior and infidelity	Mirzazadeh et al., 2018; Klimas et al., 2019; Lacker et al., 2020	
	Dishonesty and cheating in games/non-academic tasks	Höglinger and Jann, 2018; Atsusaka and Stevenson, 2020; Jensen, 2020	
	Attitudes toward refugees, Muslims, and xenophobia	Johann and Thomas, 2017; Hoffmann et al., 2020; Meisters et al., 2020b	
	Voting and voter intention	Waubert de Puiseau et al., 2017; Höglinger and Jann, 2018	
	Adherence to COVID-19 measures	Jensen, 2020; Mieth et al., 2021	
	Blood donation	Walzenbach and Hinz, 2019	
		**(CM%** **>** **DQ%) vs. True Aggregate-Level%**	**(CM%** **<** **DQ%) vs. True Aggregate-Level%**
Aggregate-level validation studies (*k* = 9)	Excessive drinking	Höglinger and Diekmann, 2017	
	Academic misconduct	Jerke et al., 2021	Jerke et al., 2021
	Health and STDs	Höglinger and Diekmann, 2017; Nasirian et al., 2018	
	Dishonesty and cheating in games/non-academic tasks	Hoffmann et al., 2015; Höglinger and Jann, 2018; Meisters et al., 2020a	Höglinger and Jann, 2018
	Islamophobia and xenophobia	Hoffmann and Musch, 2016	
	Voting intention	Lehrer et al., 2019	
	Blood and organ donation	Höglinger and Diekmann, 2017	Walzenbach and Hinz, 2019)
		**(CM%** **>** **DQ%) vs. True Individual-Level%**	**(CM%** **<** **DQ%) vs. True Individual-Level%**
Individual-level validation studies (*k* = 1)	Dishonesty and cheating in prediction and roll-a-six games		Höglinger and Jann, 2018
CM-only prevalence studies (*k* = 6)	Substance use and misuse	Khosravi et al., 2015; Kazemzadeh et al., 2016; Heck et al., 2018; Vakilian et al., 2019	
	Tax evasion	Kundt et al., 2017	
	Health and STDs	Heck et al., 2018	
	Sexual behavior	Vakilian et al., 2014, 2016; Kazemzadeh et al., 2016	
	Abortion	Eslami et al., 2013	

*Excluded from analysis/table for absence of estimates: Academic misconduct (Hoffmann et al., 2017; Jerke et al., 2019), and health (Schnapp, 2019)*.

### Sensitive/Transgressive Behavior

Overall, majority of studies (*k* = 13) investigated substance use and misuse whereas others examined academic misconduct (*k* = 8), corruption, tax evasion and theft (*k* = 7), and sexual behavior and infidelity (*k* = 6). Other studies investigated dishonesty and cheating in games/non-academic tasks (*k* = 5), attitudes toward refugees, Muslims, and xenophobia (*k* = 4), health and STDs (*k* = 4), voting and voter intention (*k* = 3), adherence to COVID-19 measures (*k* = 2), blood and organ donation (*k* = 2), and abortion (*k* = 1). See Supplementary Tables 1, 2 and Table 1 for details of the studies.

### Innocuous/Unrelated Items

Thirty-five studies used birthdays of the respondent or their family members and acquaintances, totaling 62 birthday innocuous item pairs. Sixteen studies employed non-birthday innocuous items (Kundt, 2014; Shamsipour et al., 2014; Vakilian et al., 2014, 2016, 2019; Khosravi et al., 2015; Kundt et al., 2017; Mirzazadeh et al., 2018; Nasirian et al., 2018; Banayejeddi et al., 2019; Hopp and Speil, 2019; Lehrer et al., 2019; Safiri et al., 2019; Schnapp, 2019; Atsusaka and Stevenson, 2020; Jerke et al., 2021) totaling 46 item pairs. In addition, seven studies (Shamsipour et al., 2014; Khosravi et al., 2015; Nasirian et al., 2018; Banayejeddi et al., 2019; Hopp and Speil, 2019; Safiri et al., 2019; Schnapp, 2019) used a combination of birthday and non-birthday innocuous item pairs.

Phone numbers were used in seven studies (Shamsipour et al., 2014; Khosravi et al., 2015; Vakilian et al., 2016, 2019; Kundt et al., 2017; Banayejeddi et al., 2019; Safiri et al., 2019), house numbers in seven studies (Kundt, 2014; Shamsipour et al., 2014; Khosravi et al., 2015; Lehrer et al., 2019; Safiri et al., 2019; Schnapp, 2019; Vakilian et al., 2019), ATM card pin code in three studies (Shamsipour et al., 2014; Khosravi et al., 2015; Safiri et al., 2019), and ID card number in two studies (Khosravi et al., 2015; Safiri et al., 2019). The remaining studies relied on random numbers or letters of the alphabet (Banayejeddi et al., 2019), performance of academic tasks (Jerke et al., 2021), date of a significant personal event (Hopp and Speil, 2019), family size of four (Nasirian et al., 2018), owning a vehicle (Nasirian et al., 2018), friend or family member with a common name (Vakilian et al., 2014, 2019), picking a card (Mirzazadeh et al., 2018), and random probability assignment (Atsusaka and Stevenson, 2020). The exact question was not available in one study (Kazemzadeh et al., 2016). See Supplementary Table 4.

### Participants

Samples comprised university students (*k* = 16), members of online panels (*k* = 9), general or community samples (*k* = 8), academics (*k* = 3), high school students (*k* = 2), men (*k* = 2), bodybuilders (*k* = 1), HIV patients (*k* = 1), employees (*k* = 1), postpartum women (*k* = 1), and prisoners (*k* = 1). See Supplementary Table 1.

### Sample Size

In total, the studies included about 71,278 participants (with notable sample overlap such as Shamsipour et al., 2014). Sample size ranged from 20 (Jerke et al., 2019), a qualitative study, to 15,972 (Jerke et al., 2021) and were justified by power analysis in 13 (Vakilian et al., 2014, 2016, 2019; Hoffmann et al., 2015, 2017; Khosravi et al., 2015; Heck et al., 2018; Höglinger and Jann, 2018; Banayejeddi et al., 2019; Meisters et al., 2020a,b; Canan et al., 2021; Mieth et al., 2021) of the 45 studies.

### Model Design

CM applications employed various model probabilities ranging from *P* = 0.086 (Atsusaka and Stevenson, 2020) to 0.842 (Meisters et al., 2020b). Twenty-two studies used *P* = 0.25 (a 25% expected affirmation of the innocuous item based on birthday month or season). Of these, 49 pairs used specific birthday months with P ranging between 0.08 (1/12 months) and 0.25 (3/12 months). In five cases (Eslami et al., 2013; Nakhaee et al., 2013; Khosravi et al., 2015; Nasirian et al., 2018; Safiri et al., 2019), season (e.g., spring) was used which is open for interpretation by the respondents (e.g., a birthday on March 28th could mean “meteorological winter” and “astronomical spring”). In six cases (Shamsipour et al., 2014; Khosravi et al., 2015; Heck et al., 2018; Banayejeddi et al., 2019; Safiri et al., 2019; Meisters et al., 2020b), the birthday question was ambiguous (e.g., born between certain days or months) which could be interpreted as either including or excluding the days or months.

For studies employing items with uncertain probabilities such as name of friend or relative (Vakilian et al., 2014, 2019), number of main family members and owning a vehicle (Nasirian et al., 2018), conference attendance and research proposal writing (Jerke et al., 2021), authors relied on population statistics for probabilities. The probability of a “yes” answer for the innocuous items in these studies ranged from *P* = 0.08 to *P* = 0.7, with *P* = 0.33 being most frequent. The range of sensitive items in a single study varied from one (*k* = 18) to six (*k* = 2: Banayejeddi et al., 2019; Safiri et al., 2019). In case of multiple sensitive items (*k* = 22), authors reported unique and independent estimates. Here, independency between the innocuous items was ensured in 13 studies, dependency in four studies, whereas information is not available or unclear in five studies. See Supplementary Tables 2, 4 for study details.

### Sensitive Item Framing and Timeframe

We evaluated nine studies (Eslami et al., 2013; Nakhaee et al., 2013; Kazemzadeh et al., 2016; Heck et al., 2018; Mirzazadeh et al., 2018; Nasirian et al., 2018; Hoffmann et al., 2020; Özgül, 2020; Mieth et al., 2021) as presenting sensitive items that are unclear and subject to misinterpretation. Additionally, ten studies were evaluated (Eslami et al., 2013; Nakhaee et al., 2013; Kazemzadeh et al., 2016; Heck et al., 2018; Mirzazadeh et al., 2018; Nasirian et al., 2018; Banayejeddi et al., 2019; Hopp and Speil, 2019; Jensen, 2020; Meisters et al., 2020b) as presenting sensitive items that are non-factual and judgmental. The time frames for sensitive items were diverse and spanned future, present, past 2 weeks, past month, past 12 months, past 10 years, lifetime, and unspecified periods. See Supplementary Table 2.

### Mode of Administration

Twenty-one studies administered CM using online questionnaires. CM was also administered using paper questionnaires (*k* = 18), interviews (*k* = 4), a combination of interviews and questionnaires (*k* = 2), and an unspecified questionnaire (*k* = 1) See Table 2.

**Table 2:** Mode of CM administration.

**Mode**	**References**
Online questionnaires (*k =* 21)	Korndörfer et al., 2014; Kundt, 2014; Roberts and St. John, 2014; Hoffmann et al., 2015, 2017; Höglinger et al., 2016; Höglinger and Diekmann, 2017; Waubert de Puiseau et al., 2017; Höglinger and Jann, 2018; Hopp and Speil, 2019; Klimas et al., 2019; Lehrer et al., 2019; Schnapp, 2019; Walzenbach and Hinz, 2019; Atsusaka and Stevenson, 2020; Jensen, 2020; Lacker et al., 2020; Meisters et al., 2020a; Canan et al., 2021; Jerke et al., 2021; Mieth et al., 2021
Paper questionnaires (*k =* 18)	Coutts et al., 2011; Jann et al., 2012; Nakhaee et al., 2013; Shamsipour et al., 2014; Vakilian et al., 2014, 2016, 2019; Khosravi et al., 2015; Hoffmann and Musch, 2016; Kazemzadeh et al., 2016; Heck et al., 2018; Mirzazadeh et al., 2018; Nasirian et al., 2018; Banayejeddi et al., 2019; Safiri et al., 2019; Hoffmann et al., 2020; Meisters et al., 2020b; Özgül, 2020
Interviews (*k =* 4)	Gingerich et al., 2015; Johann and Thomas, 2017; Kundt et al., 2017; Oliveros and Gingerich, 2020
Interviews and questionnaires (*k =* 2)	Eslami et al., 2013; Jerke et al., 2019
Unspecified questionnaire (*k =* 1)	Hopp and Speil, 2019

### Hypotheses and Conclusions

In a study comprising comparative, aggregate-level, and individual-level validation studies, Höglinger and Jann (2018) indicate that “more is not always better” in explaining their finding that CM estimates are sometimes affected by false positives and false negatives. It can therefore be inferred that “more is not always better” (for undesirable behavior) and conversely ‘less is not always better’ (for desirable behavior). Thirty-nine studies applied the “more is better” hypothesis. Of these, 22 affirmed the “more is better” hypothesis whereas 17 concluded that “more is not always better” due to factors such as the tendency for false positives or overreporting and non-compliance. Also, five studies used the “less is better” hypothesis with two studies affirming this hypothesis and three concluding that ‘less is not always better’ due to the propensity for false negatives or underreporting and non-compliance. See Table 3, Supplementary Table 1.

**Table 3:** Hypotheses and results/conclusion of included studies.

**Hypothesis**	**Result/Conclusion**	
**More is better (*k =* 39)**	**More is better (*k =* 22)**	**More is not always better (*k =* 17)**
Coutts et al., 2011; Jann et al., 2012; Eslami et al., 2013; Nakhaee et al., 2013; Korndörfer et al., 2014; Kundt, 2014; Roberts and St. John, 2014; Shamsipour et al., 2014; Vakilian et al., 2014, 2016, 2019; Gingerich et al., 2015; Hoffmann et al., 2015, 2017, 2020; Hoffmann and Musch, 2016; Höglinger et al., 2016; Kazemzadeh et al., 2016; Höglinger and Diekmann, 2017; Johann and Thomas, 2017; Kundt et al., 2017; Heck et al., 2018; Höglinger and Jann, 2018; Mirzazadeh et al., 2018; Nasirian et al., 2018; Hopp and Speil, 2019; Klimas et al., 2019; Lehrer et al., 2019; Safiri et al., 2019; Schnapp, 2019; Atsusaka and Stevenson, 2020; Jensen, 2020; Lacker et al., 2020; Meisters et al., 2020a,b; Oliveros and Gingerich, 2020; Özgül, 2020; Canan et al., 2021; Jerke et al., 2021	Coutts et al., 2011; Jann et al., 2012; Eslami et al., 2013; Nakhaee et al., 2013; Kundt, 2014; Roberts and St. John, 2014; Vakilian et al., 2014, 2016, 2019; Gingerich et al., 2015; Kazemzadeh et al., 2016; Kundt et al., 2017; Hopp and Speil, 2019; Klimas et al., 2019; Safiri et al., 2019; Hoffmann et al., 2020; Jensen, 2020; Lacker et al., 2020; Meisters et al., 2020b; Özgül, 2020; Canan et al., 2021; Jerke et al., 2021	Korndörfer et al., 2014; Shamsipour et al., 2014; Hoffmann et al., 2015; Khosravi et al., 2015; Hoffmann and Musch, 2016; Höglinger et al., 2016; Höglinger and Diekmann, 2017; Johann and Thomas, 2017; Waubert de Puiseau et al., 2017; Heck et al., 2018; Höglinger and Jann, 2018; Mirzazadeh et al., 2018; Nasirian et al., 2018; Lehrer et al., 2019; Atsusaka and Stevenson, 2020; Meisters et al., 2020a; Oliveros and Gingerich, 2020
**Less is better (*****k=*** **5)**	**Less is better (*****k=*** **2)**	**Less is not always better (*****k=*** **3)**
Höglinger and Diekmann, 2017; Banayejeddi et al., 2019; Schnapp, 2019; Walzenbach and Hinz, 2019; Mieth et al., 2021	Banayejeddi et al., 2019; Mieth et al., 2021	Höglinger and Diekmann, 2017; Schnapp, 2019; Walzenbach and Hinz, 2019

### Non-compliance

Motivated and unmotivated non-compliance and its effects were assessed in eight studies (Kundt, 2014; Shamsipour et al., 2014; Höglinger and Diekmann, 2017; Heck et al., 2018; Höglinger and Jann, 2018; Schnapp, 2019; Atsusaka and Stevenson, 2020; Meisters et al., 2020a). Seven studies (Kundt, 2014; Roberts and St. John, 2014; Hoffmann et al., 2015, 2020; Höglinger et al., 2016; Lehrer et al., 2019; Walzenbach and Hinz, 2019) considered non-compliance but did not report its effects.

### CM Variants

Three studies provided variants of CM (Heck et al., 2018; Schnapp, 2019; Atsusaka and Stevenson, 2020). One group of researchers (Heck et al., 2018) proposed the extended crosswise model (ECM). The ECM has been shown to be adequately powered and provides the possibility of detecting a variety of response biases. It is noteworthy that the ECM's power equals the power of the original CM (Heck et al., 2018), and the ECM has received additional empirical support (Hoffmann et al., 2020; Meisters et al., 2020b; Mieth et al., 2021). Also, an adjustment of the conventional CM for random answers at the sample (CMR-S) and individual (CMR-I) levels has been proposed (Schnapp, 2019). Similarly, a bias correction procedure and software (cWise) has been developed for CM (Atsusaka and Stevenson, 2020).

### CM Evaluation Studies

Fourteen studies evaluated CM (Kundt, 2014; Shamsipour et al., 2014; Khosravi et al., 2015; Hoffmann and Musch, 2016; Höglinger et al., 2016; Hoffmann et al., 2017; Höglinger and Diekmann, 2017; Höglinger and Jann, 2018; Banayejeddi et al., 2019; Jerke et al., 2019; Lehrer et al., 2019; Schnapp, 2019; Walzenbach and Hinz, 2019; Meisters et al., 2020a). In a study of iron supplementation among 1,740 Iranian female high school students (Banayejeddi et al., 2019), 67.3% had high or very high trust in CM's confidentiality (low or very low: 8.3%), and 72.4% had high or very high understanding of CM's instructions (low or very low: 4.7%). In addition, understanding CM's instructions was positively correlated with trust in CM's confidentiality. In a study of 1,312 German university students (Hoffmann and Musch, 2016), the estimated prevalence of the non-sensitive item from CM (46.6%) did not significantly differ from the known true prevalence (43.3%). Also, in a study of 401 German high school students (Hoffmann et al., 2017), DQ was perceived as significantly more comprehensible than CM although CM was perceived as providing significantly higher privacy protection. However, there was no significant correlation between comprehension and perceived privacy protection.

In a study of Swiss university students (Höglinger et al., 2016), the conventional question-based CM (CMq) had significantly higher break-off, item non-response, and answering time as well as lower trust in anonymity and disclosure risk compared to DQ. Particularly, of the 1,008 CMq participants, 8.6% evaluated the technique as cumbersome, 97.0% applied the technique correctly, 67.4% perceived the technique as providing privacy protection, 59.9% evaluated the technique as reasonable, and 62.2% understood the technique. In a study of a German panel (Höglinger and Diekmann, 2017), CM produced more false positives or overreporting than DQ. In a similar study of US residents (Höglinger and Jann, 2018), CM performed better than DQ in estimating the true cheating rate in one game (prediction) but worse in another (roll-a-six). Also, although CM performed significantly better than DQ in estimating the true positive rate in the prediction game, DQ had a significantly higher correct classification rate compared to CM.

Moreover, in a qualitative evaluation of CM in 20 German, Swiss, and UK academics (Jerke et al., 2019), it was found that although a majority comprehend CM instructions, many do not understand the logic and principles of CM and that there is no relationship between CM comprehension and honesty. In a study of 1,644 Iranian university students (Khosravi et al., 2015), 40.3% indicated full comprehension of CM whereas 21.6% indicated little or no comprehension. In the same study, 33.70% indicated full trust in CM whereas 26.4% indicated little or no trust, with a positive association between CM comprehension and trust. Also, in a German study involving 256 CM participants (Kundt, 2014), 63.0% indicated that they fully understood the mechanism of CM and that it provides privacy protection, 21.0% indicated that CM provides privacy protection although they did not exactly understand CM mechanism, and 16.0% had no understanding of CM.

Additionally, in a study of a German voter panel (Lehrer et al., 2019), it was found that CM has a significantly lower item non-response compared to DQ. Although CM overestimated the true prevalence by 7.4% in the same study, it performed better than DQ as CM's confidence interval covered the true estimate. In a similar study of a German panel, it has been demonstrated that the provision of detailed instructions can lead to the minimization of false positives or overreporting among highly educated persons thus underlining the importance of detailed instructions and checks for comprehension in CM applications (Meisters et al., 2020a). Moreover, in an Iranian study (Shamsipour et al., 2014), CM estimates for two non-sensitive items were almost equal to the true prevalence values. In addition, 76.0% of 1,490 CM respondents indicated that they fully understood CM instructions, 17.0% indicated that they partially understood CM instructions, whereas 7.0% did not understand CM instructions. Also, 89.0% were highly or moderately confident in CM's privacy protection with 11.0% having little or no confidence. There was also a significant positive association between understanding CM and confidence in its privacy protection, and item non-response was 1.1% for CM but 2.9% for DQ. Furthermore, in a study of 103 Germans (Schnapp, 2019), it was found that the conventional CM generates false positive estimates of 2.0, 5.0, and 21.0% and random responses ranging of 2.0, 2.0, and 6.8% on three zero prevalence diseases. Finally, in a study of a German voter panel (Walzenbach and Hinz, 2019), there was a higher number of item non-response in CM compared to DQ.

### Quality Assessment

**Figure 4:**
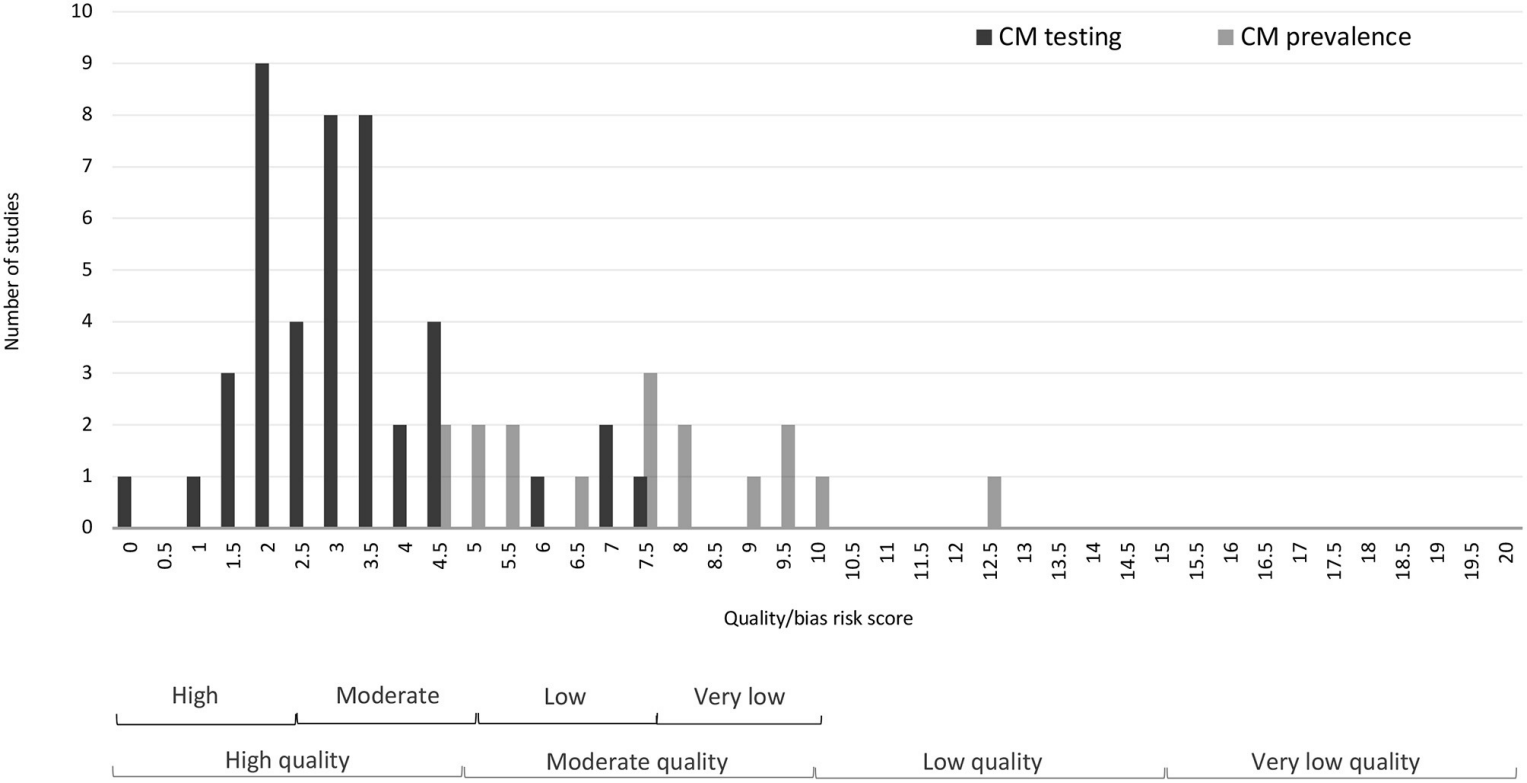
Distribution of quality and bias assessment scores for CM testing and CM prevalence studies.

The inter-reviewer reliability was found to be Fleiss' kappa = 0.66 (*p* < 0.001) indicating very good agreement between the evaluation of the lead reviewer (DS) and the final evaluation of the group (DS, OS, RC in discussion with AP). The group reached consensus on discrepant evaluations through discussion. Altogether, 11 studies were assessed as high quality/low risk, 31 were evaluated as moderate quality/risk studies, two studies were evaluated as low quality/high risk, whereas one study did not meet criteria for assessment as it was a qualitative exploration of CM. Taking a more nuanced evaluation, 18 studies were set out to establish prevalence of a specific sensitive or transgressive behavior (CM prevalence). Applying the full assessment criteria, four of the studies met criteria for high quality/low risk, 13 were assessed as moderate quality/risk, and one as low quality/high risk. The primary aim in the other 26 studies was establishing the validity of CM (CM testing), and thus a different sampling strategy was employed. Among these studies, seven were assessed as high quality/low risk, 18 as moderate quality/risk, and one as low quality/high risk. Results of the quality assessment are presented in Table 4, Figure 4, Supplementary Table 4.

**Table 4:** Summary of results of the quality assessment of included studies.

**Study type**	**Component**	**Quality assessment and studies**					
		**Low quality/high risk**	**References**	**Moderate quality/risk**	**References**	**High quality/low risk**	**References**
Overall (*K* = 44)	–	*k* = 2	Nakhaee et al., 2013; Mirzazadeh et al., 2018	*k* = 31	Coutts et al., 2011; Jann et al., 2012; Eslami et al., 2013; Korndörfer et al., 2014; Roberts and St. John, 2014; Shamsipour et al., 2014; Vakilian et al., 2014, 2016, 2019; Hoffmann et al., 2015, 2017; Hoffmann and Musch, 2016; Höglinger et al., 2016; Kazemzadeh et al., 2016; Johann and Thomas, 2017; Kundt et al., 2017; Waubert de Puiseau et al., 2017; Heck et al., 2018; Nasirian et al., 2018; Banayejeddi et al., 2019; Hopp and Speil, 2019; Klimas et al., 2019; Lehrer et al., 2019; Safiri et al., 2019; Schnapp, 2019; Walzenbach and Hinz, 2019; Jensen, 2020; Lacker et al., 2020; Meisters et al., 2020b; Özgül, 2020; Mieth et al., 2021	*k* = 11	Kundt, 2014; Gingerich et al., 2015; Khosravi et al., 2015; Höglinger and Diekmann, 2017; Höglinger and Jann, 2018; Atsusaka and Stevenson, 2020; Hoffmann et al., 2020; Meisters et al., 2020a; Oliveros and Gingerich, 2020; Canan et al., 2021; Jerke et al., 2021
CM testing (*k* = 26)	–	*k* = 1	Mirzazadeh et al., 2018	*k* = 18	Coutts et al., 2011; Jann et al., 2012; Korndörfer et al., 2014; Hoffmann et al., 2015, 2017; Hoffmann and Musch, 2016; Höglinger et al., 2016; Johann and Thomas, 2017; Kundt et al., 2017; Heck et al., 2018; Nasirian et al., 2018; Hopp and Speil, 2019; Lehrer et al., 2019; Safiri et al., 2019; Schnapp, 2019; Walzenbach and Hinz, 2019; Meisters et al., 2020b; Özgül, 2020	*k* = 7	Kundt, 2014; Höglinger and Diekmann, 2017; Höglinger and Jann, 2018; Atsusaka and Stevenson, 2020; Hoffmann et al., 2020; Meisters et al., 2020a; Jerke et al., 2021
CM prevalence (*k* =18)	Testing	*k* = 2	Nakhaee et al., 2013; Kazemzadeh et al., 2016	*k* = 9	Eslami et al., 2013; Roberts and St. John, 2014; Waubert de Puiseau et al., 2017; Banayejeddi et al., 2019; Klimas et al., 2019; Vakilian et al., 2019; Jensen, 2020; Lacker et al., 2020; Mieth et al., 2021	*k* = 7	Shamsipour et al., 2014; Vakilian et al., 2014, 2016; Gingerich et al., 2015; Khosravi et al., 2015; Oliveros and Gingerich, 2020; Canan et al., 2021
	Prevalence	*k* = 1	Nakhaee et al., 2013	*k* = 13	Eslami et al., 2013; Roberts and St. John, 2014; Shamsipour et al., 2014; Vakilian et al., 2014, 2016, 2019; Kazemzadeh et al., 2016; Waubert de Puiseau et al., 2017; Banayejeddi et al., 2019; Klimas et al., 2019; Jensen, 2020; Lacker et al., 2020; Mieth et al., 2021	*k* = 4	Gingerich et al., 2015; Khosravi et al., 2015; Oliveros and Gingerich, 2020; Canan et al., 2021

*Excluded from analysis/table: Jerke et al. (2019)—qualitative study*.

Patterns of “penalty” scores (see Table 5) provide indication of where improvements can be made. The main reason for reduced quality/increased risk of bias for the prevalence studies was representativeness of the sample (affected 89.5% of the relevant studies), followed by response rate (73.7%) and issues with the survey instrument (73.7%). Sampling affected about half (52.6%) of the studies. Among the factors affecting study quality and bias, the most salient is lack of attention to non-compliance which earned a penalty score of 72.2% of the studies. Other observed problems of CM were logged for the reliability of estimations in 63.3% of the papers, power of the analysis in 53.3% and suitability of the innocuous items in 44.4% of the cases. The results shown in Table 5 also suggest that easy improvement could be made by making the target sensitive item clear and unambiguous (17.8%), and factual (21.1%) as opposed to value-laden or judgmental.

**Table 5:** Patterns of quality and bias assessment scores.

	**Prevalence (*****k*** **=** **19)**										**Testing (*****k*** **=** **45)**									
	**1. Representation**	**2. Sampling frame**	**3. Random selection**	**4. Response rate**	**5. Primary data**	**6. Definition**	**7. Instrument**	**8. Consistency**	**9. Period**	**10. Estimation**	**11. Justification**	**12. Target clear**	**13. Target fact**	**14. Innocuous clear**	**15. Power**	**16. Non-compliance**	**17. Protection**	**18. Parameter**	**19. Estimate(s) reliable**	**20. Innocuous modeled**
Sum	17.0	10.0	10.0	14.0	0.0	1.0	14.0	0.0	5.0	0.0	4.5	8.0	9.5	4.0	24.0	32.5	0.0	11.0	28.5	20.0
%	89.5	52.6	52.6	73.7	0.0	5.3	73.7	0.0	26.3	0.0	10.0	17.8	21.1	8.9	53.3	72.2	0.0	24.4	63.3	44.4

*Maximum score for each criterion is the number of relevant papers*.

### Meta-Analysis of CVS

**Figure 5:**
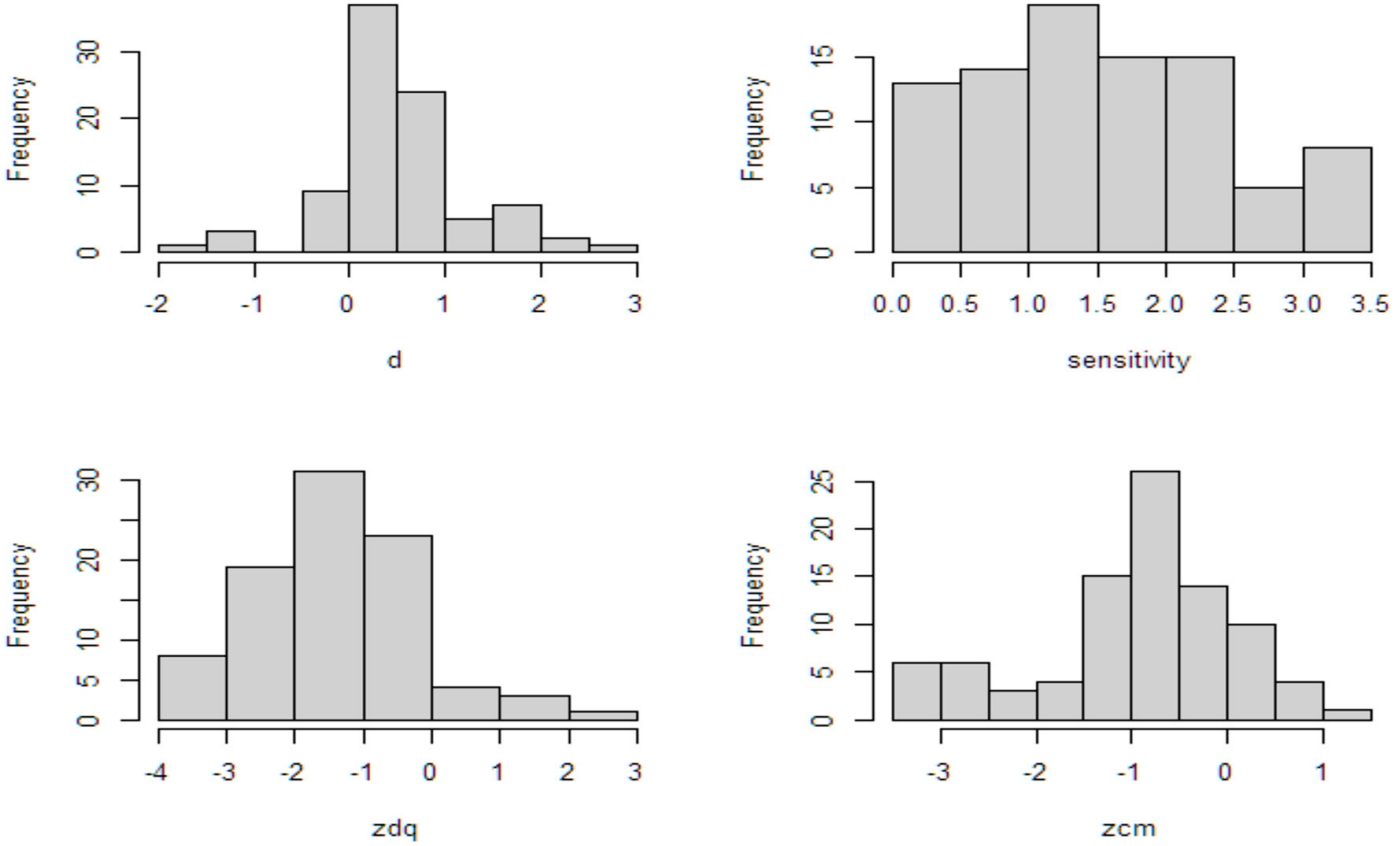
Histograms of *d*, sensitivity, and the z-scores for the DQ and CM prevalence estimates (after imputation of the infinite scores by −3.5).

Results of the calculation of *SE* as a function of probability of the sensitive behavior and probability of the affirmative answer to the innocuous item for various sample sizes are presented in Supplementary Table 6. We identified 34 CVS (DQ vs. CM) with a total of 89 items. The distributions of the *d* and sensitivity variables are depicted as histograms in Figure 5. The distribution of the *d* variable shows that CM outperforms DQ except for five items with negative scores whereas the histogram of sensitivity shows the proxy for sensitivity of the items. The intercept-only model yields an effect size of 0.49 (*SE* = 0.09, *t* = 5.21, *p* < 0.01) indicating that CM outperforms DQ by an average of 0.49 on the probit scale. With the addition of sensitivity to the model, the residual variance decreases from 0.33 to 0.27, indicating improved model fit. The slope for sensitivity shows that with each unit increase in the sensitivity of the item, the *d* score increases on average by 0.08 (*SE* = 0.15, *t* = 3.72, *p* < 0.01) on the probit scale. This indicates that the more sensitive the item, the better CM outperforms DQ. Furthermore, the M1 intercept is no longer significant indicating that for items with a DQ prevalence estimate around 50%, the difference between the DQ and CM estimates disappears. Results of the meta-analytic comparison of CM and DQ are presented in Table 6, Figure 5.

**Table 6:** Results of multilevel analytic comparison of CM and DQ.

	**M0: Intercept only (*SE*)**	**M1: Sensitivity added (*SE*)**
Intercept	0.49 (0.09)^*^	0.08 (0.15)
Sensitivity	–	0.29 (0.08)^*^
*$\sigma_{study}^{2}$*	0.14	0.17
*$\sigma_{residual}^{2}$*	0.33	0.27
Deviance	176.0	164.5

* *p < 0.01*.

### Authors' Collaboration Map

**Figure 6:**
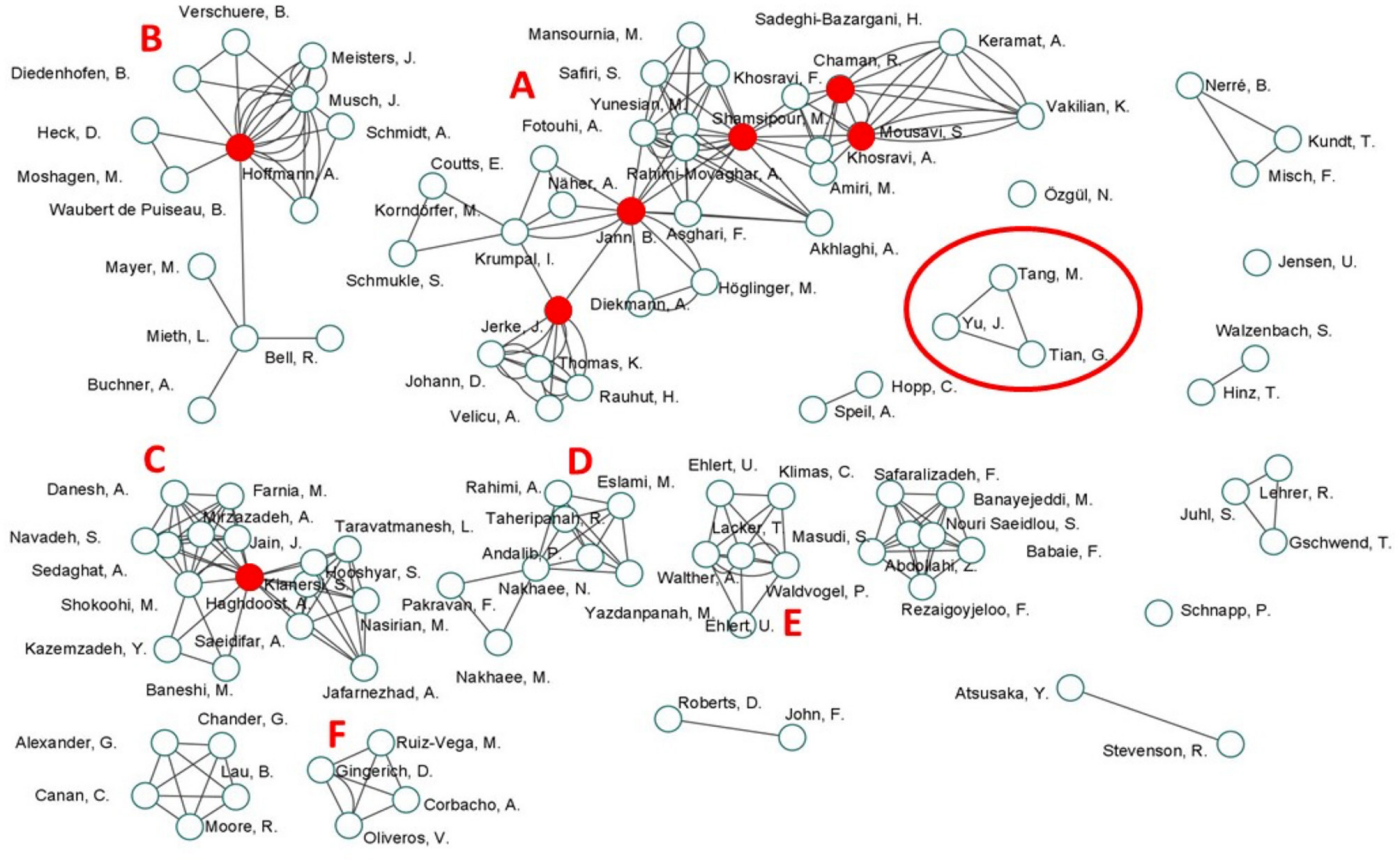
Author collaboration map based on the 45 included studies. Letters A–F denote distinct hubs. Red dots denote authors with high stress centrality values.

The 45 studies were authored by 108 researchers forming 278 connections. Network analysis through co-authorships of the included studies revealed six hubs (clusters) with multiple publications, along with a set of one-off applications of CM to investigate a variety of sensitive issues. The co-authorship map is depicted in Figure 6 with over-time changes captured in Supplementary Video 1. The co-authorship network properties, analyzed as a non-directed graph, are summarized in Supplementary Table 7.

Overall, the authors' network was moderately connected (centralization = 0.33) with three major hubs (denoted with letters “A,” “B,” and “C” in Figure 6) involving 58 (53.7%) authors who produced 57.8% of the articles (*k* = 26). The most prolific hub was “A” (*k* = 14), followed by “B” (*k* = 9), and “C” (*k* = 3). Three additional small hubs were also identified (denoted with letters “D,” “E,” and “F” in Figure 6), formed by 20 authors accounting for 18.5% of all authors and collectively producing 13.3% of the included articles (*k* = 6, two by each hub). Hub was identified if authors produced at least two articles with different authorship arrangement. The remaining 13 articles (28.9%) were one-off research endeavors and involved 30 authors (27.7%).

Eighteen authors were identified with non-zero stress value (i.e., having at least one shortest path going through them): Chaman, R., Fotouhi, A., Haghdoost, A., Hoffmann, A., Jann, B., Jerke, J., Krumpal, I., Lacker, T., Mieth, L., Mousavi, S., Musch, J., Nakhaee, N., Rahimi-Movaghar, A., Shamsipour, M., Shokoohi, M., Waldvogel, P., Walther, A., and Yunesian, M. Among these, five authors were identified as key players in the authors' network: Jann, B., Jerke, J. and Shamsipour, M. in hub A; Hoffmann, A. in hub B, and Haghdoost, A. in hub C. The summary of their node attributes are presented in Supplementary Table 8.

### Quality of Authors' Collaboration

The mean quality assessment scores did not differ [*t*_(43)_ = −0.08, *p* = 0.937] between CM testing (*M* = 3.19 ± 1.31) and CM prevalence (*M* = 3.22 ± 1.80) studies. The quality assessment however is more nuanced when examined by author cluster (see Table 7). Among the three main author hubs, clusters “A” and “B” have better quality CM testing whereas cluster “C” has better quality CM prevalence.

**Table 7:** Average quality assessment scores by authors' clusters.

		**Study type (*****k*****)**		**Score**	
**Cluster**	***k***	**CM**	**CM**	**CM testing**	**CM prevalence**
		**testing**	**prevalence**	**(max = 10)**	**(max = 20)**
A	14	8	6	2.464 ± 0.664	6.063 ± 2.382
B	9	7	2	2.670 ± 1.173	7.500 ± 0.500
C	3	2	1	6.670 ± 0.577	5.330 ± 4.509
D	2	0	2	6.000 ± 2.121	10.000 ± 3.536
E	2	0	2	4.000 ± 0.707	9.500 ± 0.000
F	2	0	2	1.750 ± 0.354	4.750 ± 0.354
Other	13	4	9	3.231 ± 0.904	6.890 ± 1.557

### Authors' Collaboration and Mode of Administration

There appeared to be a slight preference for online vs. paper-and-pencil applications between author clusters. For example, cluster “A” used more online surveys (8/14) and cluster “B” had a slight tendency toward paper-and-pencil surveys (5/9). However, there was no unique pattern of authors' mode of CM administration [χ^2^_(18)_ = 15.35, Fisher's exact *p* = 0.719]. Similarly, quality assessment scores did not differ [*F*_(2, 42)_ = 0.31, *p* = 0.738] by mode of administration.

## Discussion

We conducted a systematic review and meta-analysis as well as quality assessment of empirical applications of CM with the primary focus on the method. Specifically, we categorized and reviewed empirical application by study type, model format, mode of administration, year of publication, geographical location of the study and sample, nature of the sensitive issue, compliance and honesty, quality, and performance against DQ. Pulling together 45 studies, we distilled valuable information on what constitutes a “good CM study,” identify areas for improvement, and make recommendations for empirical applications.

### Study Origin

CM has proved useful in quantitative, qualitative as well as mixed-method studies of a variety of sensitive or transgressive behavior and samples around the world. Included studies originated from three continents with Europe leading, followed by Asia and America. However, there is limited variability in study origin with majority of European studies originating from Germany or based on German samples. Studies in Asia were exclusively conducted in Iran, whereas studies in America originate from the US and Costa Rica.

### Sensitive/Transgressive Behavior

Among the 45 studies included in this review, the sensitivity of the investigated issues varies widely but in-depth cultural and contextual understanding is needed to judge the degree of sensitivity of each. For example, taking iron supplements appears to be a non-sensitive issue in many contexts. However, CM use in the assessment of iron supplementation in an Iranian study (Banayejeddi et al., 2019) is justified because iron supplementation was mandatorily administered to high school students and not taking them is regarded a defiant act. It is also noteworthy that issues such as abortion, blood donation, or engaging in pre- and extramarital sex vary in degree of sensitivity by culture and context.

### Model Design

Birthdays with *P* ranging between 0.2 and 0.25 were the most popular choice for the innocuous item. Although birthdays are not exactly evenly distributed throughout the year, using birthdays for the unrelated innocuous item is a better choice than, for example, house numbers, having a sibling, a friend with a certain name, attending more than four scientific conferences in the last 12 months, or working on a research grant proposal. The implications of independency between the innocuous items in studies where multiple and related sensitive items were used are two-fold. On the one hand, respondents may get suspicious if the innocuous items are iterations of the same, such as mother's birthday. On the other hand, independency allows for calculating correlations between the estimates which in turn can help establish validity.

### Non-compliance

Sensitive items yield non-compliance in multiple ways (Yan, 2021). Beyond the impact on respondents' willingness to participate in the first place, refusing to answer the sensitive item (item non-response) leads to missing data, whereas the accuracy of respondents' answers to sensitive items (measurement error) impacts data validity.

Our finding that the effect of non-compliance was assessed in only eight studies is noteworthy. CM studies often encounter challenges stemming from complex instructions (in comparison to DQ), lack of trust, and the reluctance to give a seemingly compromising response (Shamsipour et al., 2014; Höglinger et al., 2016; Hoffmann et al., 2017; Höglinger and Jann, 2018; Banayejeddi et al., 2019; Jerke et al., 2019). These can lead to unmotivated non-compliance where respondents do not adhere to the instructions for reasons such as poor understanding of the instructions or carelessness. Non-compliance is also muddled with deliberate untruthful responding (Coutts et al., 2011; Hoffmann et al., 2017). Non-compliance is a problem with CM more prominently so than it is with DQ (Höglinger and Diekmann, 2017; Höglinger and Jann, 2018).

Whilst non-compliance in DQ usually emerges from self-protection, motivated and goal-oriented non-compliance is mixed with lack of attention and understanding in CM non-compliance (Coutts et al., 2011; Höglinger et al., 2016). Compliance is related to trust that the method provides protection, understanding of the instructions and motivation for honest responding (Hoffmann and Musch, 2016; Jerke et al., 2019). For efficiency, future CM studies are encouraged to use item formats that minimize non-motivated non-compliance while offering transparent protection against exposure. Relatedly, qualitative studies examining experiences of CM such as trust and understanding (Jerke et al., 2019) may elucidate further CM method and provide opportunities for further advancement of CM (Hoffmann et al., 2017).

### Authors' Collaboration and Mode of Administration

Overall, the co-authorship network from the past 10 years of empirical work using CM indicates that research has been driven by methodology and prevalence estimation roughly in equal measure. It is also notable that the proponents of CM (Yu et al., 2008), have not conducted or participated in any of the empirical applications of the model. This separation of theory and practice is characteristic of the IEM field in general. Researchers in this field tend to form three distinct groups: (1) “desktop research” focusing on method development with a mathematical and statistical orientation, (2) social science survey methodologists with interest in specific IEM performance in empirical application, and (3) epidemiologists and public health researchers with sole interest in obtaining prevalence estimates. The CM literature conforms to this pattern. In addition, there appears to be a slight preference for online vs. paper-and-pencil applications between author groups. However, this is probably driven by convenience and sample characteristics rather than methodological considerations.

### Meta-Analysis of CVS

Given the value of meta-analysis in research (Murad et al., 2016), our study provides a strong empirical indication that CM outperforms DQ and even better with increased behavior sensitivity. It is however important to treat the above evidence with caution given our additional finding of little difference between DQ and CM estimates for items with a DQ prevalence estimate around 50%. The above findings are consistent with results of the earlier meta-analysis of RRT (Lensvelt-Mulders et al., 2005a) showing that RRT lead to more valid estimates compared to DQ, and that the performance of RRT improve with increasing item sensitivity.

### Quality Assessment, and Strengths and Limitations of CM

Results of the quality assessment showing that majority of CM studies are of moderate quality indicates some weaknesses in previous empirical applications of CM, and the importance of caution in the use of and conduct of CM research. The evaluation of CM performance is improved with enhanced privacy protection, trust and comprehensibility, and the ability to disentangle false negatives and false positives (Shamsipour et al., 2014; Hoffmann et al., 2017; Höglinger and Diekmann, 2017; Höglinger and Jann, 2018; Nasirian et al., 2018; Jerke et al., 2019; Walzenbach and Hinz, 2019).

From the quality assessment, key areas of CM research requiring improvement are sampling, particularly the use of representative samples, low response rate, the use of valid and reliable measurement instruments, and the assessment of non-compliance. Given that sensitivity leads to various forms of non-compliance which threatens the validity of survey data (Yan, 2021), the widespread lack of non-compliance assessment is improper. The results of the quality assessment also suggests that CM can be improved by ensuring clear reporting of the parameters of estimates (e.g., CI and *SE*), conducting *a priori* power analysis, making the sensitive item clear, unambiguous, and factual as opposed to being value-laden or judgmental, and examining the suitability of innocuous items.

Generally speaking, IEM are more effective but less efficient than DQ (Lensvelt-Mulders et al., 2005a). The choice between the two is highly contextual. In situations where IEM are likely to yield more valid data, the loss of efficiency is compensated with a gain in effectiveness. The aim of any IEM development is keeping the loss in efficiency as small as possible to capitalize on the gain in effectiveness and make the IEM more profitable (Lensvelt-Mulders et al., 2005b). A disadvantage of IEM is that they are less efficient than DQ because IEM work by including random noise or a degree of uncertainty in non-randomized models with known or assumed distribution to the response data. This added noise inevitably leads to larger standard errors and reduced power which necessitates considerably larger samples than DQ.

The obvious advantage of IEM is the enhanced level of protection for both the respondents and the researcher. The former aims to alleviate fears of exposure and encourage honest reporting on socially sensitive or transgressive behavior (Tourangeau and Yan, 2007). The latter can be a useful feature in situations where the researcher is under legal or ethical obligation to break confidentiality and report on positive cases. Such situations could arise, for example, in anti-doping research if the researcher has reporting obligations under the World Anti-Doping Code, or in prison studies where data collection on transgressions (e.g., possessing drugs or weapons) among inmates is conducted by staff. With IEM, by making it impossible to identify “positive cases” under any circumstance, this concern is automatically removed from the study design.

CM, in comparison to other IEM, is quite advantageous in terms of efficiency. Expressing efficiency in terms of power and required sample size, Ulrich et al. (2012, p. 626) set the minimum sample size for Warner's method (and apply to the CM based on a mathematical similarity) to detect 10% prevalence with *P* = 0.3 exposure, and power of 0.95 at *N* > 1,500, which drops to *N* > 1,000 with power reduced to 0.85, and to *N* > 700 with power of 0.80. To detect 20% prevalence, *N* > 500 is sufficient. Sample size also has an impact on efficiency. Using the same scenario (*P* = 0.30, assumed 10% prevalence), figures from Supplementary Table 6 indicate that the *SE* decreases from 0.053 (*N* = 500) to 0.037 (*N* = 1,000) and 0.031 (*N* = 1,500). Using the equation of 95% CI = *SE* x 3.92 to convert *SE* to 95% CI, these figures, are 0.10 ± 0.2078, 0.1450, and 0.1228, respectively.

Additionally, results of the meta-analysis of CVS indicate that taking efficiency into account, the choice between CM and DQ should hinge on the sensitivity of the research issue or behavior. Inferably, although we provide convincing empirical evidence for the superiority of CM to DQ in terms of effectiveness, this evidence has limited generalizability. In relation to the above, although most studies relied on the “more is better” hypothesis, it has been demonstrated that this hypothesis is sometimes flawed due to the propensity for misclassification of responses, particularly false positive responding (Umesh and Peterson, 1991; Höglinger and Jann, 2018). It is therefore important that false positives as well as false negatives are taken into consideration in CM research.

### Optimizing CM

Findings from the 14 studies (Kundt, 2014; Shamsipour et al., 2014; Khosravi et al., 2015; Hoffmann and Musch, 2016; Höglinger et al., 2016; Hoffmann et al., 2017; Höglinger and Diekmann, 2017; Höglinger and Jann, 2018; Banayejeddi et al., 2019; Jerke et al., 2019; Lehrer et al., 2019; Schnapp, 2019; Walzenbach and Hinz, 2019; Meisters et al., 2020a) on how participants perceive CM format and comply with its instructions are mixed. For instance, whereas understanding CM's instructions is positively correlated with trust in CM's confidentiality (Khosravi et al., 2015; Banayejeddi et al., 2019), there is no significant correlation between comprehension and perceived privacy protection in another study (Hoffmann et al., 2017). It has also been indicated that even some highly educated persons such as academics (Jerke et al., 2019) and university students (Khosravi et al., 2015; Höglinger et al., 2016) do not understand the logic and principles of CM. Here, it is plausible that incomprehensibility of CM instructions and non-compliance hampers CM's effectiveness rather than lack of understanding of CM method itself. The pessimistic take on CM not being better than DQ (Jerke et al., 2019) diverges from the extant literature on IEM (Lensvelt-Mulders et al., 2005a).

Moreover, findings from CM evaluation studies provide sufficient evidence for the need for further optimization of CM. It is evident that most applications of CM are CVS (Hoffmann et al., 2015; Höglinger and Jann, 2018) with fewer aggregate-level and individual-level validation studies. With the three variants of CM identified in this study (Heck et al., 2018; Schnapp, 2019; Atsusaka and Stevenson, 2020), further advancements of CM method particularly including individual-level and aggregate-level validation (Höglinger and Jann, 2018) are encouraged. It is important to point out that CM addresses many of the recommendations of (Lensvelt-Mulders et al., 2005b). Unlike the Forced Response model (Boruch, 1971), CM does not force participants to answer “yes” under any condition and an affirmative response is always masked. Additionally, in comparison to the models with two-step instructions such as the Unrelated Question models (Horvitz et al., 1967; Greenberg et al., 1969, 1971; Mangat, 1994), CM features a one-step procedure with relatively simple instruction. Simplicity and fast completion in turn reduces the cognitive demand and is assumed to reduce un-motived non-compliance.

Due to the sample size required for IEM, participants' completion of both the DQ and CM format back-to-back in some studies is understandable on feasibility grounds, but the potential order effect should be mitigated. Studies which use the same sample for both CM and DQ administered the survey formats without randomization across the sample. This means that participants responded to an item about the same sensitive issue in two survey formats in a fixed order. From the cognitive point of view, it is not likely that respondents give a different answer to the sensitive item. It is however unlikely that a respondent who provides a false response about the sensitive issue in DQ format changes his/her mind and admits the same moments later in the same survey, and vice versa. This affects all studies with a crossover design at the individual level, but the impact is at least mitigated at sample level if the order is randomized. CVS using a split sample with random allocation are methodologically superior, and thus offer better evidence for the effectiveness of CM against DQ.

There is an inherent trade-off between the statistical power and protection offered by IEM. Intuitively, a high level of protection requires enough random noise to mask individual responses to the sensitive item. Ulrich et al. (2012) observe that for Warner's model, which is mathematically but not conceptually equivalent to CM, the optimal level of protection is achieved by setting the *P* of the innocuous item to 0.5 but reduces the power to 0. On the other hand, setting *P* to 0 or 1 maximizes the power but offers no privacy protection. Therefore, for the optimal balance between efficiency and effectiveness, it is recommended that in line with most studies using CM to date, exposure or protection is kept at *P* = 0.2–0.3 (or equivalently *P* = 0.7–0.8). It is vital to carefully select innocuous items where: (1) the distribution is known or could be assumed with a great degree of confidence, and (2) which is specific and not open to interpretation by the respondent. Clear and unambiguously worded instructions and items help to reduce unmotivated non-compliance (i.e., those arising from misinterpreting the items, too complex to understand, or wanting to spend the time to understand).

The recommended minimum sample size depends on the assumed prevalence of the sensitive issue or behavior in the population, and the protection and effectiveness. To facilitate determining the sample size *a priori*, we included Supplementary Table 6. Incremental improvements to reducing the 95% CI can be made by limiting the estimation for the finite sample (if this sufficiently addresses the research question) instead of estimating prevalence for the infinite population. In surveys, data quality is a function of the amount of measurement error in the data (Yan, 2021). The mechanism of giving a dishonest answer in DQ is straightforward but it is less so in some IEM. As deception requires more cognitive effort than honest responding (Gombos, 2006; Walczyk et al., 2013), IEM, such as CM, that offer no obvious option for false reporting are more advantageous. Simply put, it takes more time and effort to figure out which response option of CM is better for false reporting (i.e., hiding in the “both or none” vs. the “only one yes answer” group) than being honest under full protection.

### Strengths, Limitations, and Future Research Recommendations

Based on the 45 studies included in this review, CM has proved valuable in quantitative, qualitative as well as mixed-method studies of a variety of sensitive or transgressive behavior around the world with various samples. In terms of samples, university students comprise the predominant sample for CM studies. To our knowledge, the present study is the first to include a bespoke quality assessment of CM and IEM in general. Developed specifically for IEM with future application in mind, our design and application of a quality assessment measure for CM is also novel and another strength of our study.

During the final revision of the present article, we discovered another meta-analysis of CM (Schnell and Thomas, 2021) comprising 25 studies and 33 CVS presenting 141 estimates from the literature up to February 2020. Their results indicate that the difference between CM and DQ is 4.88. Meta-regression analysis found that for general population and non-probability samples, the difference between CM and DQ is smaller. The authors explain this finding as an education effect where the difference between CM and DQ estimates are associated with highly educated samples. They therefore question the advantage of CM over DQ in general population samples. However, differences between the DQ and CM estimates were analyzed on a probability scale, where the difference between 1 and 5% is equal to the difference of 40 and 44%. In contrast, using a probit scale as in our meta-analysis, the former difference is much larger than the latter, which makes the estimated effect size of 4.88 difficult to interpret. This is an important difference between the two meta-analyses, which can cast doubt about the interpretation and recommendation of Schnell and Thomas (2021) regarding the applicability of CM for general population samples or samples with low educational level.

It is also noteworthy that the two meta-analyses were developed parallelly. However, our meta-analysis includes almost twice as many studies as were included in Schnell and Thomas' (2021) meta-analysis. Additionally, all studies included in Schnell and Thomas' (2021) meta-analysis were included in our meta-analysis apart from one study (Corbacho et al., 2016) which is a duplicate of one included study (Gingerich et al., 2015), and four other studies (Enzmann, 2017; Enzmann et al., 2018; Gschwend et al., 2018; Schnell et al., 2019) which do not meet our language and record type inclusion criteria. Our metanalysis also has other advantages such as a more-detailed description of included studies in tables and supplementary tables, quality assessment, a mapping of authors' collaboration, and a more-detailed elucidation of the precincts and prospects of CM. Altogether, the two meta-analyses present complementary evidence on the functionality of CM and underscore the importance of refining meta-analytical techniques specific to IEM.

Although we provide reassuring empirical evidence for the superiority of CM to DQ, this evidence has limited generalizability particularly for items with a DQ prevalence estimate around 50%. Whereas neither review can make a conclusive judgement regarding educational level and suitability of CM or any IEM to that effect, among IEM, CM is relatively simple in terms of instructions and cognitive demand albeit still more complicated than DQ. Nonetheless, it is reasonable that an interplay exists between educational level, more specifically reading level, comprehension and fluidity, and the complexity in survey instructions. It is also plausible that this relationship is moderated by motivation and task engagement. Future research is required to examine and quantify this link specifically for IEM in self-report surveys. Furthermore, it is conceivable that there is a minimum threshold for reading comprehension above which educational level makes no difference.

IEM have been developed for added protection on sensitive issues. Therefore, instead of “giving up” and reverting to DQ, as may be inferable from Schnell and Thomas' (2021) finding, further research should aim at making CM and IEM in general as simple and as accessible as possible. Specifically, CM studies are encouraged to provide detailed information and include comprehension checks, use sensitive item formats that minimize non-compliance while offering transparent protection against exposure for efficiency. Moreover, qualitative studies examining experiences of CM such as trust and understanding may elucidate further CM method and provide opportunities for further advancement of CM. In addition, experimenting with graphical representation of the responses instead of, or in addition to, the written instructions and responses may be beneficial. It is also important that false positives as well as false negatives are taken into consideration in CM research. Relatedly, further aggregate-level and individual-level validation studies are encouraged in the advancement of CM method. Weaknesses in previous empirical applications of CM underline the importance of caution in the use of and conduct of CM research.

Alongside the demonstrated strengths, we also acknowledge the limitations of our study. First, we limited our literature search to articles published in English. Although there is no evidence suggesting cultural differences based on the available information (i.e., the included studies conducted in nine countries) and the broader literature on IEM, it is possible that we have missed important data and methodological developments published in languages other than English. Further potential limitations arise from the relatively small number of studies and a wide variety of parameters (e.g., the sensitive behavior or attribute, sample characteristics, sample size, randomization probability, mode of administration and the overall as well as specific quality measures) which could not be fully explored in our meta-analysis due to the small sample size in each subgroup. With the present study however, we set up a potentially useful framework for future systematic reviews and meta-analyses of CM studies as well as other widely used IEM.

The quality/bias assessment tool for IEM was developed alongside its first application, which partially explains the interrater agreement. The ten items on IEM were refined through their applications to the CM used in the included studies. Although we were mindful of the need for generalizability throughout the development process, subsequent independent application is warranted to test its applicability to other IEM. We also recognize that the cut-off points or discrete quartiles used in the quality assessment are arbitrary to some degree. However, in addition to the categorization, we provide detailed continuum scores in Supplementary Table 4 for informativeness.

Furthermore, about half of the studies included in this review administered CM via the Internet using some online survey platform, which readily offers the option to record the time taken to complete the CM survey. Future studies should consider making use of this feature and routinely reporting the average completion time to inform further empirical applications. Response time can also be exploited in experimental settings to develop better understanding of non-compliance and finding ways to differentiate between unmotivated and motivated non-compliance. CM is a promising variant of the rich collection of IEM. The method will benefit from more strong validation studies where estimated prevalence is compared to the known prevalence or can be compared to an external, independent measure of same. More comparative studies contrasting CM against other IEM are also warranted with focus on efficiency, effectiveness, and resistance to non-compliance.

## Conclusion

With a few notable exceptions, attempts to evidence validity and accuracy of the fundamental assumptions of CM, such as distribution of the unrelated innocuous item and full compliance with the instructions, are taken for granted. Many studies, assuming “more is better,” interpreted higher estimates from CM compared to DQ as indication that CM is closer to the “true prevalence” and evidence of CM's validity. Although critical evaluation is warranted for improvement, CM is a promising tool for assessing sensitive/transgressive behavior owing to its sufficient protection, flexibility, relative simplicity, and suitability for self-administration. Methodically sound application of CM requires expert input into optimizing the model design and administration. The quality assessment tool we developed for this review is suitable for any IEM and can thus help advance the field by supporting the design of future empirical studies and in applications to systematic reviews and meta-analyses on IEM.

## Data Availability Statement

The original contributions presented in the study are included in the article/Supplementary Material. Further inquiries can be directed to the corresponding author.

## Author Contributions

DS and AP designed the study, conducted the literature search and selection, and drafted the manuscript. DS, OS, RC, and AP performed the quality assessment. MC conducted the meta-analysis. All authors contributed to the writing and revision process and approved the final manuscript.

## Conflict of Interest

DS, MS, OH, and AP are members, and MC and PH are associated members of the World Anti-Doping Agency's Working Group on Doping Prevalence. They prepared the review in this capacity with support from OS and RC. Members of the Working Group receive no payment for their work but expenses directly related to the Working Group are covered by WADA. WADA has no influence over the content of this paper.
