# Supplementary material for: Functionality of the Crosswise Model for Assessing Sensitive or Transgressive Behavior: A Systematic Review and Meta-Analysis
Source: Front Psychol. 2021 Jun 23;12:655592. doi: 10.3389/fpsyg.2021.655592 (PMC8260852; doi:10.3389/fpsyg.2021.655592)
Supplement: Supplementary file 1 [file Data_Sheet_1.PDF]

SUPPLEMENTARY MATERIAL

Functionality of the Crosswise Model for Assessing Sensitive or Transgressive Behavior: A Systematic Review and Meta-Analysis

SUPPLEMENTARY TABLE 1. Empirical applications of CM to assess sensitive or transgressive behavior

1st author Y	Behavior	Study focus	Sample	*N*	Measure	Method for split sample	Hypothesis	Result (%)	(Δ) [95% CI] (±*SE*)
Atsusaka 2020	Speeding through questions, faking answers, lying to qualify for surveys	Possibility of false positives (overreporting) and false negatives (underreporting) in CM vs DQ	US paid survey takers	§DQ: 282 Speeding (CM Low: 188, CM Moderate: 270), faking (CM Low: 189, CM Moderate: 270), lying (CM Low: 192, CM Moderate: 267)	OQ	SSSO: 1:1	MIB	‡CM > DQ: Speeding (Low: 0.31 vs 0.19; Moderate: 0.31 vs 0.19), faking (Low: 0.23 vs 0.09; Moderate: 0.11 vs 0.09), lying (Low: 0.20 vs 0.09; Moderate: 0.26 vs 0.09)	Speeding (Low: ±0.04, ±0.02; Moderate: ±0.06, ±0.02), faking (Low: ±0.04, ±0.02; Moderate: ±0.06, ±0.02), lying (Low: ±0.04, ±0.02; Moderate: ±0.06, ±0.02)
Banayejeddi 2019	Iron supplementation	Comparison of CM to DQ, evaluation of CM	Iranian female high school students	CM: 1740 DQ: 440	PQ	DSR 4:1	LIB	CM > DQ: Taking ≥1 Fe pill (76.8 vs 87.9), taking all delivered Fe pills (31.3 vs 43.0)<br><br>Trust - Very low: 2.5, low: 5.8, moderate: 24.4, high: 31.6, very high: 35.7.<br><br>Understanding - Very low: 2.0, low: 2.7, moderate: 22.9, high: 31.3, very high: 41.1	Taking ≥1 Fe pill (11.1, ±1.8), taking all delivered Fe pills (11.7, ±2.6)

Canan 2021	Prescription opioid diversion	Comparison of CM to DQ	US HIV patients	CM: 313<br>DQ: 252	OQ	DS 1:1	MIB	‡CM > DQ: 15.3 vs 6.3	[10.4–20.3 vs 3.7–10.1]
Coutts 2011	Plagiarism (partial, severe)	Comparison of CM to DQ	German university students	CM: 310<br>DQ: 96	PQ	DSR 3:1	MIB	Partial: CM (22.3) > DQ (7.3).<br><br>Severe: ‡CM (1.6) > DQ (1.0)	Partial: 15.0, ±6.1<br><br>Severe: 0.6, ±5.1
Eslami 2013	Abortion	Prevalence estimation	Iranian postpartum women	CM: 2993	I & Q	-	MIB	14.4	-
Gingerich 2015	Corruption	Comparison of CM to DQ	Costa Rica community members	CM/DQ: 4200	I	SSSO	MIB	‡CM (27.0) > DQ (20.0)	7.0: CM (±3.0), DQ (±1.0)
Heck 2018	Performance enhancing substances (PES), sexually transmitted diseases (STDs)	Prevalence estimation via ECM	German university students	322	PQ	-	MIB	‡PES: 28.9, STDs: 25.2	PES: ±5.5, STDs: ±5.4
Hoffmann 2015	Cheating on anagram task	Comparison of CM to DQ	German panel members	CM: 526<br>DQ: 138	OQ	DSNI	MIB	CM (13.0) > DQ (5.1)	7.9: CM (±2.8), DQ (±1.9)
Hoffmann 2016	Islamophobia, xenophobia	Comparison of CM to DQ	German university students	CM/DQ: 1312	PQ	SSRO	MIB	CM > DQ: Islamophobia (51.6 vs 43.3), xenophobia (48.7 vs 27.0)	Islamophobia (±8.3), xenophobia (±21.7)
Hoffmann 2017	Cheating on an exam	Comprehensibility and perceived privacy protection of CM and DQ	German high school students	CM/DQ: 401	OQ	SSS (no information on order)	-	CM < DQ: Comprehension (83.4 vs 90.4).<br>CM > DQ: perceived privacy protection (0.42 vs 0.03)	Comprehension (7.0), perceived privacy protection (0.39)
Hoffmann 2020	Xenophobia, opposing further	Comparison of CM to DQ	German university students	CM/DQ: 1382 [xenophobi	PQ	SSRO	MIB	CM > DQ: xenophobia (31.65 vs 15.45),	Xenophobia (±3.32 vs ±1.67),

	refugee admissions			a (CMn: 454, DQn: 466) opposing refugees (CMn: 465, DQn: 452]				‡opposing refugees (43.56 vs 36.73)	opposing refugees (±3.38 vs ±2.27)
Höglinger 2016	Exam misconduct (copying others, using crib notes, taking drugs, plagiarism, submitting another's paper)	Comparison of two CM (question - CMq, number - CMn) to DQ	Swiss university students	CM: 1008<br>DQ: 1004	OQ	DSR 2:2 (of 6 separate groups)	MIB	Prevalences –<br>CMq > DQ: copying others (30.1, 17.9), using crib notes (18.4, 9.1), drugs for exam performance (15.3, 3.4), plagiarism (7.6, 2.9), submitting another's paper (6.1, 0.1)<br><br>Quality –<br>CMq > DQ: break-off (2.8, 1.2), item non-response (1.4, 0.6), answering time – s (150.0, 43.0)<br><br>CMq < DQ: disclosure risk (1.5, 0.1), trust in anonymity (76.4, 0.1)<br><br>CMq: cumbersome (8.6), applied correctly (97.0), protects (67.4), reasonable (59.9), understood (62.2)	Prevalences –<br>CMq vs DQ: copying others (12.8, ±3.2), using crib notes (9.3, ±3.0), drugs for exam performance (11.9, ±2.9), plagiarism (4.7, ±3.1), submitting another's paper (4.6, ±3.1)<br><br>Quality (±) –<br>CMq vs DQ: break-off (0.5, 0.3), item non-response (0.3, 0.2), answering time – s (1.9, 0.7)<br><br>CMq vs DQ: disclosure risk (1.3, 1.5), trust in anonymity (1.4, 1.3),

Höglinger 2017	Blood donation, lifetime Chagas disease, excessive drinking, organ donation, received organ	Comparison of CM to DQ	German panel members	CM: 1123<br>DQ: 562	OQ	DSR 1:2	LIB: Suffered from Chagas disease, unwilling to donate organs, ever received donated organ<br><br>MIB: Never donated blood, excessive drinking	CM > DQ: Never donated blood (51.6 vs 48.8), unwilling to donate organs (27.3 vs 22.0), ‡excessive drinking (32.7 vs 20.6)<br><br>‡CM > DQ: False positives/overreporting [Suffered from Chagas disease (4.8 vs 0.4), ever received donated organ (7.6 vs 0.0)]	Never donated blood (2.8, ±3.2), unwilling to donate organs (5.3, ±2.9), excessive drinking (12.1, ±2.9), suffered from Chagas disease - (4.4, ±1.9), ever received donated organ (7.6, ±2.0)
Höglinger 2018	Cheating in two dice games (prediction, roll-a-six), non-voting, shoplifting, tax evasion	Possibility of false positives (overreporting) and false negatives (underreporti ng) in CM vs DQ	US residents	Prediction (DQ: 387, CM 1168), roll-a-six (DQ: 382, CM: 1145), non-voting (DQ: 766, CM: 2306), shoplifting (DQ: 768, CM: 2313), tax evasion (DQ: 768, CM: 2310)	OQ	DS	MIB	CM > DQ: Cheating in prediction (15.4 vs 2.3) and roll-a-six (14.3 vs 3.9) games, shoplifting (46.4 vs 40.2), tax evasion (19.5 vs 10.0), ‡non-voting (38.1 vs 34.5)]<br><br>‡CM > DQ: Estimating true cheating rate in prediction game Δ(-11.2 vs -21.3)<br><br>‡CM < DQ: Estimating true cheating rate in	Detecting false negatives: Prediction game (13.1, ±2.2), roll-a-six game (10.4, ±2.3), shoplifting (6.2, ±2.4), tax evasion (9.5, ±1.9), non-voting (3.6, ±2.4)<br><br>‡CM > DQ: Estimating true cheating rate in prediction game ±(2.4 vs 2.5)

								roll-a-six game Δ(8.3 vs -0.5)<br><br>CM > DQ: True positive rate [prediction game (28.4 vs 9.8)]<br><br>CM < DQ: Correct classification rate [roll-a-six game (86.0 vs 97.9)]	‡CM < DQ: Estimating true cheating rate in roll-a-six game ±(2.1 vs -1.0)<br><br>CM > DQ: True positive rate [prediction game ± (5.5 vs 3.2)]<br>CM < DQ: Correct classification rate [roll-a-six game ±(2.1 vs 0.8)]
Hopp 2019<br><br><br><br>Jensen 2020	Fare dodging, plagiarism<br><br><br><br>COVID-19 social distancing, dishonesty in a prediction dice game	Comparison of CM and DQ<br><br><br><br>Comparison of CM and DQ	Austrian university students<br><br><br>US adults	CM/DQ: 144<br><br><br><br>CM: 523<br>DQ: 536	OQ<br><br><br><br>OQ	SSSO<br><br><br><br>DS 1:1	MIB<br><br><br><br>MIB	CM > DQ: Fare dodging (60.4 vs 30.8), plagiarism (58.3 vs 24.3)<br><br>CM > DQ: Social distancing (37.0 vs 30.2), dishonesty (67.4 vs 86.6)	Fare dodging (29.6, ±13.1), plagiarism (34.0, ±9.0)<br>Social distancing (±3.2 vs ±2.0), dishonesty (±3.2 vs ±1.5)
Jann 2012	Partial and severe plagiarism	Comparison of CM to DQ	Swiss, German university students	CM: 358<br>DQ: 116	PQ	DSR 3:1	MIB	CM > DQ: Partial (22.3 vs 7.3), ‡severe (1.6 vs 1.0)	Partial (15.0, ±6.1), severe (0.6, ±5.1)
Jerke 2019	Academic misconduct (authorship, data, writing)	Qualitative assessment of CM	German, Swiss, UK academics	20	I & PQ	-	–	–	–
Jerke 2021	Self-plagiarism, data manipulation	Comparison of CM to DQ	Austrian, German, Swiss academics	CM/DQ: 15972 [self-plagiarism (CMn: 3008, DQn: 3012)	OQ	DSR	MIB	‡CM > DQ: Data manipulation (9.02 vs 1.53)	Data manipulation [-2.63- 12.35] vs [0.96-2.10], Self-plagiarism [-15.01-

				data manipulation (CMn: 1792, DQn: 1765]				‡CM < DQ: Self-plagiarism (-7.06 vs 2.99),	-5.08] vs [2.38-3.60],
Johann 2017	Attitudes towards Muslims	Comparison of CM to DQ	Austrian electoral panel members	CM/DQ: 1205	I	SSSO (in two waves with time apart)	MIB	CM (60.2) ≈ DQ (59.8).<br><br>CM > DQ: European and Muslim lifestyles are compatible	0.4: CM (±2.9), DQ (±1.4)
Kazemzadeh 2016	Alcohol consumption, extra/pre-marital sex (EPMS), methamphetamine use, opium use, relationships with the opposite sex (RWOS), tramadol misuse	Prevalence estimation	Iranian medical students	553	PQ	-	MIB	Alcohol consumption (16.8), EPMS (12.4), methamphetamine use (7.2), opium use (2.2), RWOS (42.8), tramadol misuse (14.8)	Alcohol consumption [8.9, 24.6], EPMS: [6.2, 18.5], methamphetamine use [1.2, 13.1], opium use [0.0, 9.4], RWOS [34.1, 50.5], tramadol misuse [9.4, 20.1]
Khosravi 2015	Lifetime and last month illicit drug use, opium use	Prevalence estimation, comprehension and trust in CM	Iranian university students	1644	PQ	-	MIB	Lifetime illicit drug use: 19.0, last month illicit drug use: 3.5, lifetime opium use: 14.9, last month opium use: 3.0.<br><br>Comprehension - Full: 40.3, partial: 38.1, little: 12.0, no: 9.6.<br><br>Trust - Full: 33.7, partial: 39.8, poor: 18.4, no: 8.0	Lifetime illicit drug use [13.1, 24.8], last month illicit drug use [0.0, 9.1], lifetime opium use [9.1, 20.7], last month opium use [0.0, 20.7]
Klimas 2019	Infidelity	Comparison of CM to DQ	Swiss men	CM/DQ: 224	OQ	SSSO	MIB	CM (37.7) ≈ DQ (37.5)	0.2

Korndörfer 2014	Tax evasion	Comparison of CM to DQ	German online panel	CM: 862<br>DQ: 305	OQ	DSR 3:1	MIB	CM (27.8) > DQ (16.7)	11.1: CM (±3.3), DQ (±2.1)
Kundt 2014	Tax evasion	Comparison of CM to DQ	German online panel	CM: 256<br>DQ: 137	OQ	DSNI (approx 2:1)	MIB	Prevalence: CM (30.0) > DQ (14.6)<br><br>Item non-response: CM (3.3) < DQ (9.3)	Prevalence: 15.4: CM (±4.2), DQ (±3.0)<br><br>Item non-response: -6.0
Kundt 2017	Tax evasion (paying wages in cash, underreporting of sales)	Prevalence estimation	Serbian employees	422	I	-	MIB	Cash payment: 30.2, underreporting: 33.9	Cash payment: 0.06, underreporting: 0.06
Lacker 2020	Infidelity	Prevalence estimation	Swiss men	CM/DQ: 253	OQ	SSSO	MIB	‡CM (30.2) > DQ (24.5)	
Lehrer 2019	Intention to vote for right-wing populist party	Comparison of CM to DQ	German voter panel members	CM: 867<br>DQ: 2597	OQ	DSR 1:2	MIB	‡Prevalence: CM (20.0) > DQ (9.0)<br><br>Item non-response: CM (10.8) < DQ (18.5)	Prevalence: 11.0<br><br>Item non-response: -7.7
Meisters 2020a	Cheating on anagram task	Possibility of false positives (overreporting) and false negatives (underreporting) in CM vs DQ	German panel members	CM/DQ: 2713 (CM detailed: 972, CM brief: 1164, DQ: 577)	OQ	DSR	MIB	CM > DQ: CMb (30.78) > DQ (11.79); CMd (25.48) > DQ (11.79); ‡CMb vs CMd.<br><br>False positives: CMb (14.32) > DQ (2.53); CMd (13.08) > DQ (2.53); ‡CMb vs CMd.<br><br>False negatives: CMb (56.65) < CMd (65.99) < DQ (81.77).	CMb (±2.07), CMd (±2.21), DQ (±1.34)

								Random responses: DQ > CMb; DQ > CMd; ‡CMb vs CMd.<br><br>DQ > CMb < CMd: comprehensibility, confidentiality, clarity.	
Meisters 2020b	Campus Islamophobia	Comparison of extended CM (ECM) and DQ	German university students	CM/DQ: 1361 (ECM: 911<br>DQ: 450)	PQ	DSR	MIB	ECM (21.19) > DQ (10.89)	ECM (±2.23), DQ (±1.47)
Mieth 2021	COVID-19 handwashing	Comparison of ECM and DQ	General public (fluent in German) via social media	CM/DQ: 1434 (ECM: 943<br>DQ: 491)	OQ	DSR	LIB	ECM > (78.1) > DQ (94.5)	ECM (±3.0), DQ (±1.0)
Mirzazadeh 2018	HIV-related risk behavior<br>(drug use, history of HIV testing, injection drug use, sexual risk)	Comparison of CM to DQ	Iranian prisoners	CM/DQ: 265 (M: 210, F: 55)	PQ	SSSO	MIB	‡CM > DQ: Drug injection during last incarceration (M: 15.0 vs 3.8, F: 37.5 vs 1.8), extramarital sex during past 12 months (M: 17.5 vs 12.9, F: 22.5 vs 10.9), lifetime drug injection (M: 21.3 vs 15.3, F: 41.3 vs 3.6), lifetime same-sex sex (M: 20.0 vs 12.8, F: 22.5 vs 0.0), sex during last incarceration (M: 11.3 vs 3.8, F: 23.8 vs 0.0).<br><br>‡CM < DQ:<br>Lifetime drug use (M: 65.0 vs 74.2, F: 33.8 vs 47.2).	Drug injection during last incarceration (M: 11.2, F: 35.7), extramarital sex during past 12 months (M: 4.6, F: 11.6), lifetime drug injection (M: 6.0, F: 37.7), lifetime same-sex sex (M: 7.2, F: 22.5), sex during last incarceration (M: 7.5, F: 23.8). Lifetime drug use (M: -9.2, F: -13.4). Drug use during last incarceration (M: -0.6, F: 22.3).

								‡Drug use during last incarceration: CM < DQ (M: 37.5 vs 38.1), CM > DQ (F: 35.0 vs 12.7).<br><br>‡Lifetime HIV test: CM > DQ (M: 50.0 vs 46.1), CM < DQ (F: 28.8 vs 38.1)	Lifetime HIV test (M: 3.9, F: -9.3)
Nakhaee 2013	Lifetime anabolic steroid use	Comparison of CM to DQ	Iranian bodybuilders	CM/DQ: 298	PQ	SSSO	MIB	‡CM (56.8) > DQ (24.5)	32.3
Nasirian 2018	Sexually transmitted infections (STIs; genital ulcer, rectal discharge, urethral discharge, vaginal discharge)	Comparison of CM to DQ	Iranian community members	CM: 128 (M: 64, F: 64) DQ: 1090 (M: 615, F: 475)	PQ	DS	MIB	‡CM > DQ: genital ulcer (M: 53.1 vs 2.1, F: 34.9 vs 1.5), rectal discharge (M: 21.1 vs 2.1, F: 34.9 vs 1.5), urethral discharge (M: 72.3 vs 2.2), vaginal discharge (86.3 vs 32.3)	Genital ulcer (M: 51.0, F: 33.4), rectal discharge (M: 19.3, F: 31.8), urethral discharge (M: 70.1), vaginal discharge (54.0)
Oliveros 2019	Corruption (avoid paying traffic ticket, ever bribing police)	Comparison of CM and DQ	Costa Rica community members	CM/DQ: 4193	I	-	MIB	‡CM > DQ: Avoid paying traffic ticket (22.0 vs 18.0), ever bribing police (13.0 vs 9.0)	Avoid paying traffic ticket: 4.0 CM [18.0, 25.0], DQ [17.0, 19.0]; ever bribing police: 4.0 CM [10.0, 16.0], DQ [8.0, 10.0]
Özgül 2020	Illicit drug use	Comparison of CM and DQ	Turkish university students	CM: 534 DQ: 178	PQ	DSR 3:1	MIB	CM (22.6) > DQ (6.1)	16.5: CM [14.5, 30.7], DQ [3.2, 9.0]
Roberts 2014	Research misconduct (major: data fabrication, plagiarism; minor:	Comparison of CM and DQ	UK academics	CM: 98 DQ: 93	OQ	SSSO	MIB	‡CM > DQ: Data fabrication (-5.0 vs 0.0), over-selling results (13.5 vs 8.6), plagiarism (-2.4 vs 1.1), taking	Data fabrication (CM ±0.9), over-selling results (CM ±0.9), plagiarism (CM ±0.8), taking

	inappropriate co-authorship, over-selling results, taking someone's idea)							someone's idea (-15.0 vs 1.1).<br><br>‡CM < DQ: Inappropriate co-authorship (23.3 vs 29.0)	someone's idea (CM ±0.7).<br><br>Inappropriate co-authorship (CM ±1.0)
Safiri 2019	Alcohol consumption, substance use, and extramarital sex	Comparison of CM and DQ	Iranian university students	CM/ DQ: 1730	PQ	SSSO	MIB	‡CM > DQ: illicit opioids (13.46 vs 1.84), methylphenidate (33.63 vs 2.48), extramarital sex (19.36 vs 5.54).<br><br>‡CM < DQ: alcohol (4.47 vs 4.85), cannabis (0 vs 1.21), methamphetamine (0.54 vs 1.09)	Illicit opioids: CM [9.08–17.8], DQ [1.31–2.60]; methylphenidate: CM [26.79–40.46], DQ [1.84–3.33]; extramarital sex: [13.65–25.07], DQ [4.56–6.73].<br><br>Alcohol: CM [0.01–9.68], DQ [3.93–5.97]; cannabis: CM [0–0.65], DQ [0.79–1.85]; methamphetamine: CM [0–5.70], DQ [0.70–1.71]
Schnapp 2019	Lifetime Castleman disease, Chagas disease, Barth syndrome)	Possibility of false positives/ overreporting and random responses in CM	German panel members	103	OQ	-	LIB	CM false positives – Castleman disease: 5.0, Chagas disease: 2.0, Barth syndrome: 21.0<br><br>CM random responses – Castleman disease: 2.0, Chagas disease: 2.0, Barth syndrome: 6.8	

Shamsipour 2014	Illicit drug use (lifetime, last month), opium use (lifetime, last month)	Comparison of CM to DQ	Iranian medical students	CM: 1490 DQ: 1568	PQ	DS	MIB	CM > DQ: Lifetime illicit drug use (20.2 vs 3.0), lifetime opium use (13.6 vs 1.0). ‡CM > DQ: last month illicit drug use (1.5 vs 0.2), last month opium use (3.8 vs 0.0)	Lifetime illicit drug use (17.2, ±3.7), lifetime opium use (12.6, ±3.1). ‡CM > DQ: last month illicit drug use (1.3, ±3.0), last month opium use (3.8, ±3.0)
Vakilian 2014	Sexual behavior	Prevalence estimation	Iranian university students	100	PQ	-	MIB	41.0	[36.0, 53.0]
Vakilian 2016	Condom use	Prevalence estimation using CM	Iranian university students	1500 (M: 535, F: 919)	PQ	-	MIB	M: 27.0, F: 16.0	M [17.0, 38.0], F [16.0, 24.0]
Vakilian 2019	Substance use (alcohol drinking, substance use, tobacco smoking)	Prevalence estimation	Iranian university students	1500	PQ	-	MIB	Alcohol: 33.0, substance use: 7.0, tobacco: 20.0	Alcohol: [33.0, 43.0], substance use: [0.7, 13.0], tobacco: [14.0, 27.0]
Walzenbach 2019	Past 12 months' blood donation	Comparison of CM to DQ	German panel members	CM: 855 DQ: 485	OQ	DSR 2:3	LIB	CM (18.1) > DQ (11.1). Item non-response: CM (*n*: 17) > DQ (*n*: 2).	7.0
Waubert de Puiseau 2017	Intention to vote for right-wing populist party	Comparison of CM to DQ	German voter panel members	CM: 1104 DQ: 1140	OQ	DS 1:1	MIB	‡CM (11.1) > DQ (6.5)	4.6

DS: Different study sample. DSR: Different sample random split. DSNI: Different sample, no information on method. ECM: Extended crosswise model. I: Interview. LIB: Less is better. MIB: More is better. OQ: Online questionnaire. PQ: Paper questionnaire. Q: Questionnaire. SSRO: Same sample in random order. SSSO: Same sample same order. §: Personal communication. ‡: No *p* value or not significant

SUPPLEMENTARY TABLE 2. Sensitive behavior and innocuous items in empirical applications of CM

1st author Y	Sensitive behavior	Behavior item	Nonsensitive item	Independency
Atsusaka 2020	Speeding through questions, faking answers, lying to qualify for surveys	§1. When taking a survey, I never speed through questions without reading them carefully<br><br>2. When taking a survey, I never makeup answers I know are not true<br><br>3. I have never lied to qualify for a survey for which I did not meet the qualification criteria"	Random assignment to:<br>1. 2. 3. (a). Low auxiliary probability (P = 0.086)<br><br>1. 2. 3. (b). Moderate auxiliary probability (P = 0.25)	Independent
Banayejeddi 2019	Iron (Fe) supplementation	1. Has at least one iron pill been delivered to you by the school officials, during the implementation of the iron supplementation programme?<br><br>2. Has an iron pill been delivered to you weekly and regularly by the school officials, from the beginning of the current school year?<br><br>3. Have you consumed at least one iron pill you received from school officials during the implementation of the iron supplementation programme?<br><br>4. Have you consumed all the iron pills delivered to you by the school officials during the implementation of the iron supplementation programme?<br><br>5. Have you received at least one training session about the importance of appropriate nutrition from the school officials during the implementation of iron supplementation programme?<br><br>6. Have you received regular training sessions from the school officials about the importance of appropriate nutrition simultaneously with the	1. Take one of your friends or relatives whose birthday you remember. Is his (her) birthday in March? (P = .0833)<br><br>2. Take one of your friends or relatives whose birthday you remember. Is his (her) birthday between the 1st and 3rd of the month? (P = .1)<br><br>3. Take one of the numbers 0 to 9 and do not change it. Is the number 8 your choice? (P = .1)<br><br>4. Take the phone number of your friends or relatives you know and do not change that. Is the first digit of the phone number 4? (P = .1)<br><br>5. Take one letter of the groups of eight letters from the Persian alphabet and do not change that. Does the selected letter belong to the second group? (P = .125)<br><br>6. Take the cell phone number of one of your friends or relatives you know and do not change that. Is the last digit of the cell phone number 6? (P = .1)	Unclear

		distribution of iron supplements during the implementation of the programme?		
Canan 2021	Prescription opioid diversion	Have you ever given away or sold your prescription pain medication (such as oxycodone, Percocet, tramadol)? This includes giving the medicine to a family member or friend for free	Was your mother born in February, April or November? If you do not know your mother's birthday, think of your grandmother or some other woman whose birthday you know (P = .235)	Not applicable
Coutts 2011	Plagiarism (partial, severe)	1. Partial: When writing an assignment (e.g. seminar paper, term paper, thesis), have you ever intentionally adopted a passage from someone else's work without citing the original?<br><br>2. Severe: Did you ever have someone else write a large part of an assignment for you or hand in someone else's work (e.g. from www.hausarbeiten.de) as your own?	1. Is your mother's birthday in January, February or March? (P = .25)<br><br>2. Is your father's birthday in October, November or December? (P = .25)	Independent
Eslami 2013	Abortion	History of induced abortion	Were you born in spring?	Not applicable
Gingerich 2015	Corruption	In order to avoid paying a traffic ticket, I would be willing to pay a bribe to a police officer	My mother was born in October, November, or December	Not applicable
Heck 2018	Performance enhancing substances, sexually transmitted diseases	1. Did you ever consume a performance-enhancing substance (e.g., Ritalin)?<br><br>2. Did you ever have a sexually transmitted disease?	1. Is your mother's birthday between May and July? (P = .25)<br><br>2. Is your father's birthday between August and April? (P = .75)	Independent
Hoffmann 2015	Cheating on anagram task	On the anagram task, I claimed that I had solved more anagrams than I had actually solved	I was born in November or December (P = .158)	Not applicable
Hoffmann 2016	Islamophobia, xenophobia	1. The construction of minarets should be prohibited in Germany.<br><br>2. I would mind if my daughter had a relationship with a Turkish man.	My father was born in November or December (P = .158)	Not independent
Hoffmann 2017	Cheating on an exam	I have cheated on an exam	I was born in November or December (P = .17)	Not applicable
Hoffmann 2020	Xenophobia, opposing further	1. I would mind if my 20-year-old daughter had a relationship with a Turkish man	1. My father was born in November or December (P = .158)	Not applicable

	refugee admissions	2. Germany has already received more than enough refugees	2. My mother was born in November or December (P =.158)	
Höglinger 2016	Exam misconduct (copying others, using crib notes, taking drugs, plagiarism, submitting another's paper)	1. In your studies, have you ever copied from other students during an exam?<br><br>2. In your studies, have you ever used illicit crib notes in an exam (including notes on mobile phones, calculators or similar)?<br><br>3. In your studies, have you ever used prescription drugs to enhance your performance in an exam?<br><br>4. In your studies, have you ever handed in a paper containing a passage intentionally adopted from someone else's work without citing the original?<br><br>5. In your studies, have you ever had someone else write a large part of a submitted paper for you or have you handed in someone else's paper as your own?	1. Is your mother's birthday in January, February, or March? (P = .25)<br><br>2. Is your father's birthday in October, November, or December? (P = .25)	Not independent
Höglinger 2017	Blood donation, lifetime Chagas disease, excessive drinking, organ donation, received organ	1. Have you ever donated blood?<br><br>2. Are you willing to donate your organs or tissues after death?<br><br>3. In the last two weeks, have you had five or more drinks in a row (a drink is a glass of wine, a bottle of beer, etc.)?<br><br>4. Have you ever received a donated organ (kidney, heart, part of a lung or liver, pancreas)?<br><br>5. Have you ever suffered from Chagas disease (Trypanosomiasis)?	Is your mother's birthday in January, February, or March? (P = .25)<br><br>Is your father's birthday in October, November, or December? (P = .25)	Not independent
Höglinger 2018	Cheating on two dice games	1. Have you ever intentionally taken something from a store without paying for it?	Is your mother's birthday in January, February, or March? (P = .25)	Unclear

	(prediction, roll-a-six), non-voting, shoplifting, tax evasion	2. Have you ever provided misleading or incorrect information on your tax return?<br><br>3. Did you vote in the 2012 US presidential election?<br><br>4. In the $2 dice task at the beginning of this survey: Did you honestly report whether your prediction of the dice roll was right?<br><br>5. In the $2 dice game at the beginning of this survey: Did you honestly report whether you actually rolled a 6?	Is your father's birthday in October, November, or December? (P = .25)	
Hopp 2019	Fare dodging, plagiarism	1. Are you dodging the obligatory television and radio licence fee, even though you should pay?<br><br>2. Did you use illicit resources during an exam or test?	1. Does your matriculation number end in 1, 2, or 3? (P = .333)<br><br>2. Does your birthday fall in the first quarter of the year? (P = .24816)	Independent
Jensen 2020	COVID-19 social distancing, dishonesty in a prediction dice game	1. Have you at one or more times during the past four days left your house/apartment for a non-essential purpose?<br><br>2. In the dice game, did you always honestly report whether your prediction of the dice roll was right?	1. Is your father's birthday in January or February? (if you don't know please use the birthday of another family member or a good friend) (P = .158)<br><br>2. Is your mother's birthday in January or February? (if you don't know please use the birthday of another family member or a good friend) (P = .158)	Independent
Jann 2012	Partial and severe plagiarism	1. Partial: When writing an assignment (e.g., seminar paper, term paper, thesis), have you ever intentionally adopted a passage from someone else's work without citing the original?<br><br>2. Severe: Did you ever have someone else write a large part of an assignment for you or hand in someone else's work (e.g., from www.hausarbeiten.de) as your own?	1. Is your mother's birthday in January, February, or March? (P = .25)<br><br>2. Is your father's birthday in October, November, or December? (P = .25)	Independent
Jerke 2019	Academic misconduct	–	–	Not applicable

	(authorship, data, writing)			
Jerke 2021	Self-plagiarism, data manipulation	1. I have submitted the same results to two or more journals without indicating this.<br><br>2. I have intentionally manipulated empirical data to confirm my research question.	1. In the last 12 months, I have attended more than four conferences (P = .3325)<br><br>2. In the last 12 months, I've worked on at least one research proposal (P = .7074)	Independent
Johann 2017	Attitudes towards Muslims	If you think of your living arrangements, would you prefer a neighbourhood where no Muslims live?	Is your mother's birthday in January, February, or March? (P = .25)	Not applicable
Kazemzadeh 2016	Alcohol consumption, extra/pre-marital sex (EPMS), methamphetamine use, opium use, relationships with the opposite sex (RWOS), tramadol misuse	–	–	Not available
Khosravi 2015	Illicit drug use, opium use	1. Have you over the last month used one of the narcotic drugs (cannabis, opium, opium extract, crack, heroin, glass, ecstasy)?<br><br>2. Have you used opium during the last month?<br><br>3. Have you ever used any narcotic drug (cannabis, opium, opium extract, crack, heroin, glass, ecstasy)?<br><br>4. Have you ever used opium or burned opium?	1. Think of the password of one of your ATM cards, which you use more often. Is the final figure of this password one of the numbers 5, 6, or 7?<br><br>2. Is the final figure of your ID number 2, 4, or 6? Were you born in summer? Think of the number of your father's or an acquaintance's house and do not change it. Is the rightmost figure 2, 4, or 6?<br><br>3. Think of a friend or acquaintance whom you know very well and know when exactly he was born. Is his or her birthday between the first and the tenth day of the month?<br><br>4. Think of one of your friends that you know his/her mobile number. Is the rightmost figure of his/her mobile number 2, 4 or 6?	Independent

Klimas 2019	Infidelity	Considering your current relationship, have you ever had sexual contacts to a person outside of this partnership without prior agreement of your partner?	Is your mother's birthday in January or February? (P = .167)	Not applicable
Korndörfer 2014	Tax evasion	In the past 10 years, have you made false statements on your tax return in order to pay less?	Is your mother's birthday in January, February, or March? (P = .2471)	Not applicable
Kundt 2014	Tax evasion	Have you ever intentionally underdeclared income and/or made false statements to the tax office in order to pay less or no income taxes?	Is the first digit of your of your friend's house-number 7, 8, or 9? (P = .141)	Not applicable
Kundt 2017	Tax evasion (paying wages in cash, underreporting of sales)	Firms often struggle to meet all tax obligations which impose a significant burden on firms. According to your experience and judgement, do firms like this underreport at least annual 10% of annual sales to STA for VAT and/or profit tax?	Is the last digit of your best friends phone number/of the number of the person you call most often 0 or 1? (P = .2)	Not applicable
Lacker 2020	Infidelity	During your current relationship, have you ever had sexual contacts with people outside of this relationship that were not agreed upon with your partner?	'Whether the respondents' mother was born in January or February' (P = .166)	Not applicable
Lehrer 2019	Intention to vote for right-wing populist party	Are you going to vote for the A*f*D [Alternative for Germany] in the next federal election with your second vote?	We asked you to think of a friend or relative whose house number is known to you. Is that house number's first digit a 1, 2, 3 or a 4? (P = .7)	Not applicable
Meisters 2020a	Cheating on anagram task	On the anagram task, I claimed that I had solved more anagrams than I had actually solved	I was born in November or December (P = .158)	Not applicable
Meisters 2020b	Campus Islamophobia	Many Muslim students behave in misogynist ways	1. My father was born in November or December (P = .158)<br><br>2. My father was born between January and October (P = .842)	Not applicable
Mieth 2021	COVID-19 handwashing	Do you wash your hands regularly and sufficiently long (at least 20 seconds) with soap and water?	1. Is your mother's birthday in May, June or July? (P = .242)	Not applicable
Mirzazadeh 2018	HIV-related risk behaviours (drug use, history of HIV testing, injection drug use, sexual risk)	–	Pick a card with yes/no (P = .1)	Not available

Nakhaee 2013	Lifetime anabolic steroid use	Lifetime anabolic steroid use	Is your birth in spring?	Not applicable
Nasirian 2018	Sexually transmitted infections (STIs)	Genital ulcer<br>Rectal discharge<br>Vaginal discharge<br>Urethral discharge	1. Was he/she born in a special solar month?<br><br>2. Is the number of the main family members four?<br><br>3. Do his/her main family members own vehicles (car, motorcycle)?<br><br>4. Do his/her main family members own vehicles (car, motorcycle)?	Independent
Oliveros 2019	Corruption (avoid paying traffic ticket, ever bribing police)	In order to avoid paying a traffic ticket, I would be willing to pay a bribe to a police officer.<br>I have paid, at least once, a bribe to a police officer to avoid a traffic ticket	My mother was born in October, November, or December (P = .264)	Not independent
Özgül 2020	Illicit drug use	Have you ever tried illicit drug in your lifetime?	Is your mother's birthday in January, February, or March? (P = .2471)	Not applicable
Roberts 2014	Research misconduct (major: data fabrication, plagiarism; minor: inappropriate co-authorship, over-selling results, taking someone's idea)	Have you ever:<br>1. plagiarised other peoples' work?<br><br>2. over-sold the results of a paper?<br><br>3. fabricated data and then published the results?<br><br>4. been a co-author on a paper when you have done little to warrant inclusion?<br><br>5. taken a research idea you heard at a meeting or conference and published it as your own?	1. Is your birthday in January, March or April? (P = .247)<br><br>2. Is your birthday in August, November or December? (P = .249)<br><br>3. Is your birthday in January, April or September? (P = .250)<br><br>4. Is your birthday in June, August or December? (P = .252)<br><br>5. Is your birthday in February, June or November? (P = .241)	Independent
Safiri 2019	Alcohol consumption, substance use, and extramarital sex	1. Over the past year, have you consumed alcoholic beverages at least once a month, almost every month?	1. Think of your parents' or someone else's house number and do not change it. Is the left side digit 5, 6, or 7? (P_u = .300)	Unclear

		2. Over the past year, have you used opium (either burned or its residue), or heroin, or crack at least once?<br><br>3. Have you consumed cannabis at least once over the last year?<br><br>4. Have you consumed methamphetamine at least once over the last year?<br><br>5. Have you consumed methylphenidate (Ritalin) at least once over the last year?<br><br>6. Have you had extramarital sexual affairs with the opposite gender at least once over the last year? Sexual affairs meaning copulation and intercourse leading to sexual satisfaction.	2. Please think of a friend whose birth date you know, and do not change it. Is his/her birthday in spring? (P_u = .250)<br><br>3. Think of the pin number of one of your ATM cards that you use more often: is the left side digit 5, 7, or 8? (P_u = .30)<br><br>4. Think of the ID number of a friend or acquaintance: is the right side digit 6, 7, or 8? (P_u = .3)<br><br>5. Please think of the birth date of you're a friend or acquaintance whose birth date you know precisely and do not change it: is the day between the first and tenth of a month? (P_u = .329)<br><br>6. Please think of a friend or acquaintance whose mobile number you know and do not change it: is the right side digit of the number 7, 8, or 9? (P_u = .3)	
Schnapp 2019	Lifetime Castleman disease, Chagas disease, Barth syndrome)	1. Have you ever been diagnosed with Castleman disease?<br><br>2. Have you ever been diagnosed with Chagas disease (a.k.a. American trypanosomiasis)?<br><br>3. Have you ever been diagnosed with Barth syndrome?	1. Was your mother born in January or February? (P = .167)<br><br>2. Was your father born in January or February? (P = .167)<br><br>3. Please think of your main residence. Is the first digit of your house number 8 or 9? (P = .088)	Independent
Shamsipour 2014	Illicit drug use (lifetime, last month), opium use (lifetime, last month)	1. Have you ever used any illicit drug (cannabis, opium, opium residue, crack, heroin, ice, or ecstasy) in your lifetime?<br><br>2. Have you used any illicit drug (cannabis, opium, opium residue, crack, heroin, ice, or ecstasy) during the last month?	1. Take one of your friends or acquaintances whose birthday you remember. Is their birthday between the 1st and10th of the month? (P = .3285)<br><br>2. Take the pin code of one of your ATM cards that you use frequently. Is the last digit 5, 6, or 7? (P = .3)	Independent

		3. Have you ever used either opium or its residue in your lifetime?<br><br>4. Have you used either opium or its residue during the last month?	3. Take one of your friends or acquaintances whose cell phone number you know by heart. Is the last digit 1, 2, or 3? (P = .3)<br><br>4. Take the street number of your parents or one of your acquaintances, and do not change it. Is the last digit of this number 2, 4, or 6? (P = .3)	
Vakilian 2014	Sexual behavior	Have you ever had sexual relationship (intercourse)?	Do you have any friend or relative named Ali or Mohammad?	Not applicable
Vakilian 2016	Condom use	Have you ever used a condom to prevent pregnancy?	Consider one of your friends or acquaintances that you know his/her cell phone number, is the last digit of his/her phone one of the figures 1–3? (P = .25)	Not applicable
Vakilian 2019	Substance use (alcohol drinking, substance use, tobacco smoking)	1. Have you ever used drugs?<br><br>2. Have you ever drunk alcohol?<br><br>3. Have you ever smoked cigarettes	1. Please recall the cell phone number of one of your friends or acquaintances. Is the last digit 1, 2 or 3? (P = .24)<br><br>2. Please think about one of your female friends or acquaintances, is her name Fateme, Zahra, or Maryam (either singularly or in combination with other names)? (P = .24)<br><br>3. Please recall the number of your fathers (or one of your acquaintances) house. Is the right digit 2, 4 or 6? (P = .24)	Independent
Walzenbach 2019	Past 12 months' blood donation	Have you donated blood in the past 12 months?	Is your father's birthday in January, February or March? (P = .5)	Not applicable
Waubert de Puiseau 2017	Intention to vote for right-wing populist party	I am going to vote for the A*f*D [Alternative for Germany]	I was born in November or December (P = .158)	Not applicable

§: Personal communication. P values are as reported in the included paper

SUPPLEMENTARY TABLE 3. Quality/risk of bias assessment criteria for empirical applications of the Crosswise Model.

For all items, enter 1 if information is not reported.

Item		Quality/Risk of Bias Level	Score
**1***	**Was the study's target population a close representation of the national population in relation to relevant variables? The target population refers to the group of people or entities to which the results of the study will be generalized.**	YES (LOW RISK) – The study was a national survey and the sample was drawn from a list that included all individuals in the population.	0
		NO (HIGH RISK) – The study was conducted in one province only, and it is not clear if this was representative of the national population.<br>NO (HIGH RISK) – The study was undertaken in one area only and it is clear this was not representative of the national population.	1
**2***	**Was the sampling frame a true or close representation of the target population? The sampling frame is a list of the sampling units in the target population and the study sample is drawn from this list.**	YES (LOW RISK) – The sampling frame was a list of almost every individual within the target population.<br>YES (LOW RISK) – The cluster sampling method was used, and the sample of clusters/areas was drawn from a list of all areas in the target population.	0
		NO (HIGH RISK) – The sampling frame was a list of a particular group within the overall target population, which comprised many groups.	1
**3***	**Was some form of random selection used to select the sample, OR was a census undertaken? A census collects information from every unit in the sampling frame. In a survey, only part of the sampling frame is sampled. In these instances, random selection of the sample helps minimize study bias.**	YES (LOW RISK) – The sample was selected using simple random sampling.<br>YES (LOW RISK) – The target population was one area and every person in the area was sampled.	0
		NO (HIGH RISK) – The nearest areas to the capital city were selected in order to save on cost.	1
**4***	**Was the likelihood of non-response bias minimal?**	YES (LOW RISK) – The response rate for the study was >/=75%, OR an analysis was performed that showed no significant difference in relevant demographic characteristics between responders and non-responders.<br>YES (LOW RISK) – The response rate was 68%; however, the researchers did an analysis and found no significant difference between responders and non-responders in terms of age, sex, occupation and socioeconomic status.	0

		NO (HIGH RISK) – The response rate was <75%, and if any analysis comparing responders and non-responders was done, it showed a significant difference in relevant demographic characteristics between responders.<br>NO (HIGH RISK) – The response rate was 65% and the researchers did NOT carry out an analysis to compare relevant demographic characteristics between responders and non-responders.<br>No (HIGH RISK) – The response rate was 69% and the researchers did an analysis and found a significant difference in age, sex and socio-economic status between responders and non-responders.	1
5*	**Were data collected directly from the respondents (as opposed to a proxy)? A proxy is a representative of the subject.**	YES (LOW RISK) – All eligible respondents were questioned directly.	0
		NO (HIGH RISK) – A representative of the respondent was questioned.	1
6*	**Was an acceptable case definition used in the study?**	YES (LOW RISK) – The case definition used is acceptable.	0
		NO (HIGH RISK) – The case definition used is unacceptable.	1
7*	**Was the study instrument that measured the parameter of interest shown to have reliability and validity (if necessary)?**	YES (LOW RISK) – The study instrument has been shown to have reliability and validity (if this was necessary), such as test-retest, piloting, validation in a previous study, etc.	0
		NO (HIGH RISK) – The study instrument has NOT been shown to have reliability or validity (if this was necessary).<br>NO (HIGH RISK) – The authors developed their own questionnaire and did not test this for validity or reliability.	1
8*	**Was the same mode of data collection used for all respondents? The mode of data collection is the method used for collecting information from the subjects. The most common modes are face-to face interviews, telephone interviews and self-administered questionnaires.**	YES (LOW RISK) – All eligible respondents had a face-to-face interview etc.	0
		NO (HIGH RISK) – Some respondents were interviewed over the telephone and some filled in postal questionnaires.	1
9*	**Was the length of the shortest prevalence period for the parameter of interest appropriate? The longer the prevalence period, the greater the likelihood of the respondent forgetting if they experienced the variable of interest.**	YES (LOW RISK) – Respondents were asked about the variable of interest over the past week.	0
		NO (HIGH RISK) – Respondents were asked about the variable of interest over the past three years.	1
10*	**Were the numerator(s) and denominator(s) for the parameter of interest appropriate? There may be errors in the calculation and/or reporting of the numerator and/or denominator.**	YES (LOW RISK) – There were no errors in the reporting of the numerator(s) AND denominator(s) for the prevalence of the variable of interest.	0

		NO (HIGH RISK) – There was an error in the reporting of the numerator(s) AND denominator(s) for the prevalence of the variable of interest.	1
**11**	**Does the issue justify using an indirect estimation model?**	YES (LOW RISK) – The issue is sensitive, and it is unlikely that people would be willing to answer honestly if asked directly.	0
		NO (HIGH RISK) – The issue is not sensitive and thus would be better suited for direct questioning.	1
**12**	**Is the target item clear and free of potential misinterpretation?**	YES (LOW RISK) – The target item presents the issue of interest clearly, leaving no room for misunderstanding or misinterpretation.	0
		PARTLY (MEDIUM RISK) – The target item omits an important factor (e.g., timeframe, or condition under which the issue is transgressive).	0.5
		NO (HIGH RISK) – The target item does not present the issue of interest clearly, leaving room for misunderstanding or misinterpretation.	1
**13**	**Is the target item factual and free of value judgement?**	YES (LOW RISK) – The target item is presented in a factual and non-judgemental manner.	0
		NO (HIGH RISK) – The target item includes a value judgement of the issue.	1
**14**	**Is the innocuous item clear and free of potential misinterpretation?**<br><br>***Enter zero if not applicable.***	YES (LOW RISK) – The innocuous item is clear, leaving no room for misunderstanding or misinterpretation.	0
		NO (HIGH RISK) – The innocuous item is not clear, leaving room for misunderstanding or misinterpretation.	1
**15**	**Is a power calculation conducted to justify the sample size?**	YES (LOW RISK) – Power calculation is conducted to justify sampling	0
		NO BUT ACCEPTABLE (MEDIUM RISK) – Power calculation was not conducted but the sample size is sufficient to obtain the expected distribution from the innocuous item or randomization device.	0.5
		NO (HIGH RISK) – Power calculation is not conducted, and sample size is relatively small.	1
**16**	**Is noncompliance assessed or considered?**	YES (LOW RISK) – Noncompliance is assessed, and its effects are reported.	0
		PARTLY (MEDIUM RISK) – Noncompliance is considered but its effects are not reported.	0.5

		NO (HIGH RISK) – Noncompliance is not considered.	1
**17**	**Is there an obvious self-protective strategy (e.g., saying no regardless)?**	YES (HIGH RISK) – An option for self-protective noncompliance is clearly present (e.g., option to say ‘no’).	1
		NO (LOW RISK) – There is no obvious option for self-protective noncompliance.	0
**18**	**Is a parameter [confidence interval (CI) or standard error (*SE*)] for the estimate clearly reported?**	YES (LOW RISK) – CI or *SE* is clearly reported.	0
		NO (HIGH RISK) – CI or *SE* is not clearly reported.	1
**19**	**Are the estimates reliable?**<br>**95% CI = *SE* x 3.92**	HIGH RISK – 95%CI of the prevalence estimates are higher than ±25% of the prevalence estimate; or not reported.	1
		MEDIUM RISK – 95%CI of the prevalence estimates are up to ±25% of the prevalence estimate.	0.5
		LOW RISK – 95%CI of the prevalence estimates are up to ±10%.	0
**20**	**Is the distribution of the affirmative answer to the innocuous item added to the established model?**<br><br>***Enter zero if not applicable.***	YES (LOW RISK) – The distribution is known or expected from a truly random selection (e.g., random number selection, dice roll).	0
		YES (MEDIUM RISK) – The distribution is not random, but it has been assessed and carefully considered (e.g., birthdays).	0.5
		NO (HIGH RISK) – The distribution is based on proxy indicators (e.g., percentage of households with a car).	1
**Overall quality/risk of bias:**<br>CM testing studies (Items 11–20).<br>*If score = 1, 1/10 = 10%: High quality/low risk.*<br>CM prevalence studies (Items 1–20).<br>*If score = 1, 1/20 = 5%: High quality/low risk.*		**High Quality/Low Risk**	**<25%**
		**Moderate Quality/Risk**	**25–50%**
		**Low Quality/High Risk**	**51–75%**
		**Very Low Quality/Very High Risk**	**>75%**

*Adapted from: Hoy, D., Brooks, P., Woolf, A., Blyth, F., March, L., Bain, C., ... & Buchbinder, R. (2012). Assessing risk of bias in prevalence studies: Modification of an existing tool and evidence of interrater agreement. *Journal of Clinical Epidemiology, 65,* 934–939.
Avoid duplication if combined with other assessment criteria.

SUPPLEMENTARY TABLE 4. Results of the quality assessment of included studies

		Prevalence										Testing										Testing (/10)		Prevalence (/20)	
**1st author year**	**Aim**	**1. Representation**	**2. Sampling frame**	**3. Random selection**	**4. Response rate**	**5. Primary data**	**6. Definition**	**7. Instrument**	**8. Consistency**	**9. Period**	**10. Estimation**	**11. Justification**	**12. Target clear**	**13. Target fact**	**14. Innocuous clear**	**15. Power**	**16. Noncompliance**	**17. Protection**	**18. Parameter**	**19. Estimate(s) reliable**	**20. Innocuous modeled**	**CM Score**	**Category**	**Total Score**	**Category**
Atsusaka 2020	T	NA	NA	NA	NA	NA	NA	NA	NA	NA	NA	0	0	0	0	1	0	0	0	0	0	**1**	**HQ/LR**	**-**	**-**
Banayejeddi 2019	P	1	0	0	0	0	0	0	0	0	0	1	0	1	0	0	1	0	0	1	0.5	**4.5**	**MQR**	**5.5**	**MQR**
Canan 2021	P	1	0	0	0	0	0	1	0	1	0	0	0	0	0	0	1	0	0	0	0.5	**1.5**	**HQ/LR**	**4.5**	**HQ/LR**
Coutts 2011	T	NA	NA	NA	NA	NA	NA	NA	NA	NA	NA	0	0	0	0	1	1	0	1	1	0	**4**	**MQR**	**-**	**-**
Eslami 2013	P	0	0	0	0	0	1	1	0	0	0	0	1	1	0	0	1	0	1	1	0.5	**4.5**	**MQR**	**7.5**	**MQR**
Gingerich 2015	P	1	0	0	1	0	0	1	0	0	0	0	0	0	0	0.5	1	0	0	0	0	**1.5**	**HQ/LR**	**4.5**	**HQ/LR**
Heck 2018	T	NA	NA	NA	NA	NA	NA	NA	NA	NA	NA	0	1	1	0	0	0	0	0	1	0.5	**3.5**	**MQR**	**-**	**-**
Hoffmann 2015	T	NA	NA	NA	NA	NA	NA	NA	NA	NA	NA	1	0	0	0	0.5	0.5	0	0	0.5	0.5	**3**	**MQR**	**-**	**-**
Hoffmann 2016	T	NA	NA	NA	NA	NA	NA	NA	NA	NA	NA	0	0	0	0	0.5	1	0	0	1	0.5	**3**	**MQR**	**-**	**-**
Hoffmann 2017	T	NA	NA	NA	NA	NA	NA	NA	NA	NA	NA	0	0	0	0	0	1	0	1	1	0.5	**3.5**	**MQR**	**-**	**-**
Hoffmann 2020	T	NA	NA	NA	NA	NA	NA	NA	NA	NA	NA	0	0.5	0	0	0.5	0.5	0	0	0	0.5	**2**	**HQ/LR**	**-**	**-**
Höglinger 2016	T	NA	NA	NA	NA	NA	NA	NA	NA	NA	NA	0	0	0	0	0.5	0.5	0	0	1	0.5	**2.5**	**MQR**	**-**	**-**
Höglinger 2017	T	NA	NA	NA	NA	NA	NA	NA	NA	NA	NA	0	0	0	0	0.5	0	0	0	1	0.5	**2**	**HQ/LR**	**-**	**-**

Höglinger 2018	T	NA	NA	NA	NA	NA	NA	NA	NA	NA	NA	0	0	0	0	0.5	0	0	0	1	0.5	**2**	**HQ/LR**	**-**	**-**
Hopp 2019	T	NA	NA	NA	NA	NA	NA	NA	NA	NA	NA	0	0	1	0	1	1	0	0	1	0.5	**4.5**	**MQR**	**-**	**-**
Jann 2012	T	NA	NA	NA	NA	NA	NA	NA	NA	NA	NA	0	0	0	0	1	1	0	0	1	0.5	**3.5**	**MQR**	**-**	**-**
Jensen 2020	P	1	1	1	1	0	0	1	0	0	0	0	0	0.5	0	0.5	1	0	0	0	0.5	**2.5**	**MQR**	**7.5**	**MQR**
Jerke 2021	T	NA	NA	NA	NA	NA	NA	NA	NA	NA	NA	0	0	0	0	0.5	1	0	0	0	0.5	**2**	**HQ/LR**	**-**	**-**
Jerke 2019	T	NA	NA	NA	NA	NA	NA	NA	NA	NA	NA	NA	NA	NA	NA	NA	NA	NA	NA	NA	NA	**NA**	**NA**	**NA**	**NA**
Johann 2017	T	NA	NA	NA	NA	NA	NA	NA	NA	NA	NA	0	0	0	0	0.5	1	0	0	1	0.5	**3**	**MQR**	**-**	**-**
Kazemzadeh 2016	P	1	0	0	1	0	0	0	0	1	0	0	1	1	1	1	1	0	0	1	1	**7**	**LQ/HR**	**10**	**MQR**
Khosravi 2015	P	1	0	0	1	0	0	1	0	0	0	0	0	0	0	0	1	0	0	1	0	**2**	**HQ/LR**	**5**	**HQ/LR**
Klimas 2019	P	1	1	1	1	0	0	1	0	0	0	0	0	0	0	1	1	0	1	1	0.5	**3.5**	**MQR**	**9.5**	**MQR**
Korndörfer 2014	T	NA	NA	NA	NA	NA	NA	NA	NA	NA	NA	0	0	0	0	0.5	1	0	0	1	0.5	**3**	**MQR**	**-**	**-**
Kundt 2014	T	NA	NA	NA	NA	NA	NA	NA	NA	NA	NA	0	0	0	0	1	0	0	0	1	0	**2**	**HQ/LR**	**-**	**-**
Kundt 2017	T	NA	NA	NA	NA	NA	NA	NA	NA	NA	NA	0.5	0	0	0	0.5	0.5	0	1	1	0	**3.5**	**MQR**	**-**	**-**
Lacker 2020	P	1	1	1	1	0	0	1	0	0	0	0	0	0	0	1	1	0	1	1	0.5	**4.5**	**MQR**	**9.5**	**MQR**
Lehrer 2019	T	NA	NA	NA	NA	NA	NA	NA	NA	NA	NA	0	0	0	0	0.5	0.5	0	1	1	0	**3**	**MQR**	**-**	**-**
Meisters 2020a	T	NA	NA	NA	NA	NA	NA	NA	NA	NA	NA	0	0	0	0	0	0	0	0	0	0.5	**0**	**HQ/LR**	**-**	**-**
Meisters 2020b	T	NA	NA	NA	NA	NA	NA	NA	NA	NA	NA	0	0	1	0	0	1	0	0	0	0.5	**2.5**	**MQR**	**-**	**-**
Mieth 2021	P	1	1	1	1	0	0	1	0	0	0	0	1	0	0	0	1	0	0	0	0.5	**2.5**	**MQR**	**7.5**	**MQR**
Mirzazadeh 2018	T	NA	NA	NA	NA	NA	NA	NA	NA	NA	NA	0	1	1	1	1	1	0	0	1	1	**7**	**LQ/HR**	**-**	**-**
Nakhaee 2013	P	1	1	1	1	0	0	0	0	1	0	1	1	1	0	1	1	0	1	1	0.5	**7.5**	**LQ/HR**	**12.5**	**LQ/HR**
Nasirian 2018	T	NA	NA	NA	NA	NA	NA	NA	NA	NA	NA	0	1	1	1	1	1	0	0	0	1	**6**	**MQR**	**-**	**-**
Oliveros 2019	P	1	0	0	1	0	0	1	0	0	0	0	0	0	0	0.5	1	0	0	0	0.5	**2**	**HQ/LR**	**5**	**HQ/LR**

Özgül 2020	T	NA	NA	NA	NA	NA	NA	NA	NA	NA	NA	0	0.5	0	0	0.5	1	0	0	1	0.5	**3.5**	**MQR**	**-**	**-**
Roberts 2014	P	1	1	0	1	0	0	1	0	1	0	0	0	0	0	1	0.5	0	0	1	0.5	**3**	**MQR**	**8**	**MQR**
Safiri 2019	T	NA	NA	NA	NA	NA	NA	NA	NA	NA	NA	0	0	0	1	0.5	1	0	0	0	0.5	**3**	**MQR**	**-**	**-**
Schnapp 2019	T	NA	NA	NA	NA	NA	NA	NA	NA	NA	NA	0	0	0	0	1	0	0	1	1	0.5	**3.5**	**MQR**	**-**	**-**
Shamsipour 2014	P	1	1	1	1	0	0	1	0	0	0	0	0	0	0	0.5	0	0	0	1	0.5	**2**	**HQ/LR**	**7**	**MQR**
Vakilian 2014	P	1	1	1	0	0	0	1	0	0	0	0	0	0	0	0.5	1	0	0	0	1	**2**	**HQ/LR**	**6.5**	**MQR**
Vakilian 2016	P	1	1	1	1	0	0	0	0	0	0	0	0	0	0	0.5	1	0	0	0	0	**1.5**	**HQ/LR**	**5.5**	**MQR**
Vakilian 2019	P	1	1	1	1	0	0	1	0	1	0	0	0	0	0	0.5	1	0	0	1	0.5	**3**	**MQR**	**9**	**MQR**
Walzenbach 2019	T	NA	NA	NA	NA	NA	NA	NA	NA	NA	NA	1	0	0	0	0.5	0.5	0	1	0	0.5	**3.5**	**MQR**	**-**	**-**
Waubert de Puiseau 2017	P	1	0	1	1	0	0	1	0	0	0	0	0	0	0	0.5	1	0	1	1	0.5	**4**	**MQR**	**8**	**MQR**

HQ/LR: High quality/low risk. LQ/HR: Low quality/high risk. MQR: Moderate quality/risk. NA: Not applicable. P: CM prevalence study. T: CM testing study.

Overall quality/risk of bias:

CM testing studies (Items 11–20).

If score = 1, 1/10 = 10%: High quality/low risk.

CM prevalence studies (Items 1–20).

If score = 1, 1/20 = 5%: High quality/low risk.

High quality/low risk	<25%
Moderate quality/risk	25–50%
Low quality/high risk	51–75%
Very low quality/very high risk	>75%

SUPPLEMENTARY TABLE 5. Characteristics of studies included in the meta-analysis

1st author year	Nonsensitive item	P	DQ?	DQ *N*	Q Dir	CM %	CM SE	DQ %
Atsusaka 2020	Low auxiliary probability (P = 0.086) – "Naive CM"	0.086	1	282	0	0.31	0.04	0.19
Atsusaka 2020	Moderate auxiliary probability (P = 0.25) - Naive CM	0.25	1	282	0	0.31	0.06	0.19
Atsusaka 2020	Low auxiliary probability (P = 0.086) - Naive CM	0.086	1	282	0	0.23	0.04	0.09
Atsusaka 2020	Moderate auxiliary probability (P = 0.25) - Naive CM	0.25	1	282	0	0.11	0.06	0.09
Atsusaka 2020	Low auxiliary probability (P = 0.086) - Naive CM	0.086	1	282	0	0.2	0.04	0.09
Atsusaka 2020	Moderate auxiliary probability (P = 0.25) - Naive CM	0.25	1	282	0	0.26	0.06	0.09
Banayejeddi 2019	Take one of your friends or relatives whose birthday you remember. Is his (her) birthday in March? (P = .0833)	0.0833	1	440	1	77.9	1.3	99.6
Banayejeddi 2019	Take one of your friends or relatives whose birthday you remember. Is his (her) birthday between the 1st and 3rd of the month? (P = .1)	0.1	1	440	1	22.5	1.3	73.2
Banayejeddi 2019	Take one of the numbers 0 to 9 and do not change it. Is the number 8 your choice? (P = .1)	0.1	1	440	1	76.3	1.4	87.9
Banayejeddi 2019	Take the phone number of your friends or relatives you know and do not change that. Is the first digit of the phone number 4? (P = .1)	0.1	1	440	1	31.3	1.4	43
Banayejeddi 2019	Take one letter of the groups of eight letters from the Persian alphabet and do not change that. Does the selected letter belong to the second group? (P = .125)	0.125	1	440	1	63.9	1.6	62.5
Banayejeddi 2019	Take the cell phone number of one of your friends or relatives you know and do not change that. Is the last digit of the cell phone number 6? (P = .1)	0.1	1	440	1	13.8	1.2	27.6
Canan 2021	Was your mother born in February, April or November? If you do not know your mother's birthday, think of your grandmother or some other woman whose birthday you know (P = .235)	0.235	1	313	0	15.3		6.3
Coutts 2011	Is your mother's birthday in January, February or March? (P = .25)	0.25	1	96	0	22.3		7.3
Coutts 2011	Is your father's birthday in October, November or December? (P = .25)	0.25	1	96	0	1.6		1
Eslami 2013	Were you born in spring? (P = .25)	0.25	0		0	14.4		
Gingerich 2015	My mother was born in October, November, or December (P = .25)	0.25	1	4200	0	27	3	20
Heck 2018	Is your mother's birthday between May and July? (P = .25)	0.25	0		0	28.9	5.5	
Heck 2018	Is your father's birthday between August and April? (P = .75)	0.75	0		0	25.2	5.4	
Hoffmann 2015	I was born in November or December (P = .158)	0.158	1	138	0	13	2.8	5.1
Hoffmann 2016	My father was born in November or December (P = .158)	0.158	1	138	0	51.6		43.3

Hoffmann 2016	My father was born in November or December (P = .158)	0.158	1	1312	0	48.7		27
Hoffmann 2020	My father was born in November or December (P = .158)	0.158	1	466	0	31.65	3.32	15.45
Hoffmann 2020	My mother was born in November or December (P =.158)	0.158	1	452	0	43.56	3.38	36.73
Höglinger 2016	Is your mother's birthday in January, February, or March? (P = .25)	0.25	1	1004	0	30.1		17.9
Höglinger 2016	Is your father's birthday in October, November, or December? (P = .25)	0.25	1	1004	0	18.4		9.1
Höglinger 2016	Is your mother's birthday in January, February, or March? (P = .25)	0.25	1	1004	0	15.3		15.3
Höglinger 2016	Is your father's birthday in October, November, or December? (P = .25)	0.25	1	1004	0	7.6		2.9
Höglinger 2016	Is your mother's birthday in January, February, or March? (P = .25)	0.25	1	1004	0	6.1		0.1
Höglinger 2017	Is your mother's birthday in January, February, or March? (P = .25)	0.25	1	562	0	51.6		48.8
Höglinger 2017	Is your father's birthday in October, November, or December? (P = .25)	0.25	1	562	0	27.3		22
Höglinger 2017	Is your mother's birthday in January, February, or March? (P = .25)	0.25	1	562	0	32.7		20.6
Höglinger 2017	Is your father's birthday in October, November, or December? (P = .25)	0.25	1	562	0	7.6		0
Höglinger 2017	Is your mother's birthday in January, February, or March? (P = .25)	0.25	1	562	0	4.8		0.4
Höglinger 2018	Is your mother's birthday in January, February, or March? (P = .25)	0.25	1	387	0	15.4		2.3
Höglinger 2018	Is your father's birthday in October, November, or December? (P = .25)	0.25	1	382	0	14.3		3.9
Höglinger 2018	Is your mother's birthday in January, February, or March? (P = .25)	0.25	1	768	0	46.4		40.2
Höglinger 2018	Is your father's birthday in October, November, or December? (P = .25)	0.25	1	768	0	19.5		10
Höglinger 2018	Is your mother's birthday in January, February, or March? (P = .25)	0.25	1	766	0	38.1		34.5
Hopp 2019	Does your matriculation number end in 1, 2, or 3? (P = .333)	0.333	1	144	0	60.4		30.8
Hopp 2019	Does your birthday fall in the first quarter of the year? (P = .24816)	0.25	1	144	0	58.3		24.3
Jann 2012	Is your mother's birthday in January, February, or March? (P = .25)	0.25	1	116	0	22.3		7.3
Jann 2012	Is your father's birthday in October, November, or December? (P = .25)	0.25	1	116	0	1.6		1
Jensen 2020	Is your father's birthday in January or February? (if you don't know please use the birthday of another family member or a good friend) (P = .158)	0.158	1	536	0	37	3.2	30.2

Jensen 2020	Is your mother's birthday in January or February? (if you don't know please use the birthday of another family member or a good friend) (P = .158)	0.158	1	536	1	67.4	3.2	86.6
Jerke 2021§	In the last 12 months, I have attended more than four conferences (P = .3325)	0.3325	1	3012	0	0		2.99
Jerke 2021	In the last 12 months, I’ve worked on at least one research proposal (P = .7074)	0.7074	1	1765	0	9.02		1.53
Johann 2017	Is your mother’s birthday in January, February, or March? (P = .25)	0.25	1	1205	0	60.2	2.9	59.8
Khosravi 2015	Think of the password of one of your ATM cards, which you use more often. Is the final figure of this password one of the numbers 5, 6, or 7?	0.333	0		0	19		
Khosravi 2015	Is the final figure of your ID number 2, 4, or 6? Were you born in summer? Think of the number of your father’s or an acquaintance’s house and do not change it. Is the rightmost figure 2, 4, or 6?	0.333	0		0	3.5		
Khosravi 2015	Think of a friend or acquaintance whom you know very well and know when exactly he was born. Is his or her birthday between the first and the tenth day of the month?	0.333	0		0	14.9		
Khosravi 2015	Think of one of your friends that you know his/her mobile number. Is the rightmost figure of his/her mobile number 2, 4 or 6?	0.333	0		0	3		
Klimas 2019	Is your mother’s birthday in January or February? (P = .167)	0.167	1	224	0	37.7		37.5
Korndörfer 2014	Is your mother’s birthday in January, February, or March? (P = .2471)	0.25	1	305	0	27.8	3.3	2.1
Kundt 2014	Is the first digit of your of your friend’s house-number 7, 8, or 9? (P = .141)	0.141	1	137	0	30	4.2	14.6
Kundt 2017	Is the last digit of your best friends phone number/of the number of the person you call most often 0 or 1? (P = .2)	0.2	0		0	30.2		
Kundt 2017	Is the last digit of your best friends phone number/of the number of the person you call most often 0 or 1? (P = .2)	0.2	0		0	33.9		
Lacker 2020	Whether the respondents’ mother was born in January or February' (P = .166)	0.166	1	253	0	30.2		24.5
Lehrer 2019	We asked you to think of a friend or relative whose house number is known to you. Is that house number's first digit a 1, 2, 3 or a 4? (P = .7)	0.7	1	2597	0	20		9
Meisters 2020a	I was born in November or December (P = .158)	0.158	1	577	0	30.78	2.07	11.79
Meisters 2020a	My father was born in November or December (P = .158)	0.158	1	577	0	25.48	2.21	11.79
Meisters 2020b	My father was born between January and October (P = .842)	0.842	1	450	0	21.19	2.23	10.89
Mieth 2021	Is your mother’s birthday in May, June or July? (P = .242)	0.242	1	491	1	78.1	3	94.5
Mirzazadeh 2018	Pick a card with yes/no (9/10 and 1/10, P = .1)	0.1	1	265	0	58.9		68.7
Mirzazadeh 2018	Pick a card with yes/no (9/10 and 1/10, P = .1)	0.1	1	265	0	25.7		12.8

Mirzazadeh 2018	Pick a card with yes/no (9/10 and 1/10, P = .1)	0.1	1	265	0	37		32.8
Mirzazadeh 2018	Pick a card with yes/no (9/10 and 1/10, P = .1)	0.1	1	265	0	20		3.4
Mirzazadeh 2018	Pick a card with yes/no (9/10 and 1/10, P = .1)	0.1	1	265	0	18.5		12.5
Mirzazadeh 2018	Pick a card with yes/no (9/10 and 1/10, P = .1)	0.1	1	265	0	20.4		10.2
Mirzazadeh 2018	Pick a card with yes/no (9/10 and 1/10, P = .1)	0.1	1	265	0	14		3
Mirzazadeh 2018	Pick a card with yes/no (9/10 and 1/10, P = .1)	0.1	1	265	1	45.7		44.5
Nakhaee 2013	Is your birth in spring? (P = .25)	0.25	1	298	0	56.8		24.5
Nasiriran 2018	Was he/she born in a special solar month? (P = .25)	0.25	1	615	0	53.1		2.1
Nasiriran 2018	Was he/she born in a special solar month? (P = .25)	0.25	1	475	0	34.9		1.5
Nasiriran 2018	Is the number of the main family members four? (P = .31)	0.31	1	615	0	21.1		2.1
Nasiriran 2018	Is the number of the main family members four? (P = .31)	0.31	1	475	0	34.9		1.5
Nasiriran 2018	Do his/her main family members own vehicles (car, motorcycle)? (P = .22)	0.22	1	615	0	72.3		2.2
Nasiriran 2018	Do his/her main family members own vehicles (car, motorcycle)? (P = .22)	0.22	1	475	0	86.3		32.3
Oliveros 2019	My mother was born in October, November, or December (P = .264)	0.25	1	4193	0	22		18
Oliveros 2019	My mother was born in October, November, or December (P = .264)	0.25	1	4193	0	13		9
Özgül 2020	Is your mother’s birthday in January, February, or March? (P = .2471)	0.25	1	178	0	22.6		6.1
Roberts 2014§	Is your birthday in January, March or April? (P = .247)	0.25	1	93	0	0		1.1
Roberts 2014	Is your birthday in August, November or December? (P = .249)	0.25	1	93	0	13.5		8.6
Roberts 2014§	Is your birthday in January, April or September? (P = .250)	0.25	1	93	0	0		0
Roberts 2014	Is your birthday in June, August or December? (P = .252)	0.25	1	93	0	23.3	1	29
Roberts 2014§	Is your birthday in February, June or November? (P = .241)	0.25	1	93	0	0		1.1
Safiri 2019	Think of your parents’ or someone else’s house number and do not change it. Is the left side digit 5, 6, or 7? (Pu = .300)	0.3	1	1730	0	4.47		4.85
Safiri 2019	Please think of a friend whose birth date you know, and do not change it. Is his/her birthday in spring? (Pu = .250)	0.25	1	1730	0	13.46		1.84
Safiri 2019	Think of the pin number of one of your ATM cards that you use more often: is the left side digit 5, 7, or 8? (Pu = .30)	0.3	1	1730	0	0		1.21

Safiri 2019	Think of the ID number of a friend or acquaintance: is the right side digit 6, 7, or 8? (Pu = .3)	0.3	1	1730	0	0.54	1.09
Safiri 2019	Please think of the birth date of you're a friend or acquaintance whose birth date you know precisely and do not change it: is the day between the first and tenth of a month? (Pu = .329)	0.329	1	1730	0	33.63	2.48
Safiri 2019	Please think of a friend or acquaintance whose mobile number you know and do not change it: is the right side digit of the number 7, 8, or 9? (Pu = .3)	0.3	1	1730	0	19.36	5.54
Schnapp 2019	Was your mother born in January or February? (P = .167)	0.167	0		0	5	
Schnapp 2019	Was your father born in January or February? (P = .167)	0.167	0		0	2	
Schnapp 2019	Please think of your main residence. Is the first digit of your house number 8 or 9? (P = .088)	0.088	0		0	21	
Shamsipour 2014	Take one of your friends or acquaintances whose birthday you remember. Is their birthday between the 1st and 10th of the month? (P = .3285)	0.33	1	1568	0	20.2	3
Shamsipour 2014	Take the pin code of one of your ATM cards that you use frequently. Is the last digit 5, 6, or 7? (P = .33)	0.33	1	1568	0	1.5	0.2
Shamsipour 2014	Take one of your friends or acquaintances whose cell phone number you know by heart. Is the last digit 1, 2, or 3? (P = .33)	0.33	1	1568	0	13.6	1
Shamsipour 2014	Take the street number of your parents or one of your acquaintances, and do not change it. Is the last digit of this number 2, 4, or 6? (P = .33)	0.33	1	1568	0	3.8	0
Vakilian 2014	Do you have any friend or relative named Ali or Mohammad? (P = 0.24)	0.24	0		0	41	
Vakilian 2016	Consider one of your friends or acquaintances that you know his/her cell phone number, is the last digit of his/her phone one of the figures 1–3? (P = .25)	0.25	0		0	27	
Vakilian 2016	Consider one of your friends or acquaintances that you know his/her cell phone number, is the last digit of his/her phone one of the figures 1–3? (P = .25)	0.25	0		0	16	
Vakilian 2019	Please recall the cell phone number of one of your friends or acquaintances. Is the last digit 1, 2 or 3? (P = .33)	0.33	0		0	33	
Vakilian 2019	Please think about one of your female friends or acquaintances, is her name Fateme, Zahra, or Maryam (either singularly or in combination with other names)? (P = .24)	0.24	0		0	7	
Vakilian 2019	Please recall the number of your fathers (or one of your acquaintances) house. Is the right digit 2, 4 or 6? (P = .24)	0.33	0		0	20	
Walzenbach 2019	Is your father's birthday in January, February or March? (P = .5)	0.25	1	485	1	18.1	11.1
Waubert de Puiseau 2017	I was born in November or December (P = .158)	0.166	1	1140	0	11.6	6.5

DQ? – DQ estimate available?: 0 - no, 1 - yes. Q Dir – Question or item direction: 0 - undesirable, 1 - desirable. §Negative prevalence estimates truncated at 0.

SUPPLEMENTARY TABLE 6. Standard errors (*SE*) of CM for the various combinations of sample size assumed prevalence and probability of yes (P) to the innocuous item

N	**pi**	**P = 0.10**	**P = 0.15**	**P = 0.20**	**P = 0.25**	**P = 0.30**	**P = 0.35**	**P = 0.40**
**100**	**0.05**	0.043	0.055	0.070	0.089	0.117	0.160	0.246
	**0.10**	0.048	0.059	0.073	0.092	0.118	0.162	0.247
	**0.15**	0.052	0.062	0.076	0.094	0.120	0.163	0.248
	**0.20**	0.055	0.065	0.078	0.095	0.121	0.164	0.248
	**0.25**	0.057	0.067	0.079	0.097	0.122	0.165	0.249
	**0.30**	0.059	0.069	0.081	0.098	0.123	0.165	0.249
	**0.50**	0.062	0.071	0.083	0.100	0.125	0.167	0.250
**200**	**0.05**	0.031	0.039	0.050	0.063	0.082	0.113	0.174
	**0.10**	0.034	0.042	0.052	0.065	0.084	0.114	0.174
	**0.15**	0.037	0.044	0.053	0.066	0.085	0.115	0.175
	**0.20**	0.039	0.046	0.055	0.067	0.086	0.116	0.175
	**0.25**	0.041	0.047	0.056	0.068	0.087	0.117	0.176
	**0.30**	0.042	0.048	0.057	0.069	0.087	0.117	0.176
	**0.50**	0.044	0.051	0.059	0.071	0.088	0.118	0.177
**300**	**0.05**	0.025	0.032	0.040	0.052	0.067	0.093	0.142
	**0.10**	0.028	0.034	0.042	0.053	0.068	0.093	0.142
	**0.15**	0.030	0.036	0.044	0.054	0.069	0.094	0.143
	**0.20**	0.032	0.037	0.045	0.055	0.070	0.095	0.143
	**0.25**	0.033	0.039	0.046	0.056	0.071	0.095	0.144
	**0.30**	0.034	0.040	0.047	0.057	0.071	0.096	0.144
	**0.50**	0.036	0.041	0.048	0.058	0.072	0.096	0.144
**400**	**0.05**	0.022	0.028	0.035	0.045	0.058	0.080	0.123
	**0.10**	0.024	0.030	0.037	0.046	0.059	0.081	0.123
	**0.15**	0.026	0.031	0.038	0.047	0.060	0.081	0.124
	**0.20**	0.027	0.032	0.039	0.048	0.061	0.082	0.124
	**0.25**	0.029	0.033	0.040	0.048	0.061	0.082	0.124
	**0.30**	0.030	0.034	0.040	0.049	0.062	0.083	0.125
	**0.50**	0.031	0.036	0.042	0.050	0.063	0.083	0.125
**500**	**0.05**	0.019	0.025	0.031	0.040	0.052	0.072	0.110
	**0.10**	0.021	0.026	0.033	0.041	0.053	0.072	0.110
	**0.15**	0.023	0.028	0.034	0.042	0.054	0.073	0.111
	**0.20**	0.025	0.029	0.035	0.043	0.054	0.073	0.111
	**0.25**	0.026	0.030	0.036	0.043	0.055	0.074	0.111
	**0.30**	0.026	0.031	0.036	0.044	0.055	0.074	0.111
	**0.50**	0.028	0.032	0.037	0.045	0.056	0.075	0.112
**1000**	**0.05**	0.014	0.018	0.022	0.028	0.037	0.051	0.078
	**0.10**	0.015	0.019	0.023	0.029	0.037	0.051	0.078
	**0.15**	0.016	0.020	0.024	0.030	0.038	0.052	0.078
	**0.20**	0.017	0.020	0.025	0.030	0.038	0.052	0.078
	**0.25**	0.018	0.021	0.025	0.031	0.039	0.052	0.079
	**0.30**	0.019	0.022	0.026	0.031	0.039	0.052	0.079
	**0.50**	0.020	0.023	0.026	0.032	0.040	0.053	0.079
**1500**	**0.05**	0.011	0.014	0.018	0.023	0.030	0.041	0.063
	**0.10**	0.012	0.015	0.019	0.024	0.031	0.042	0.064
	**0.15**	0.013	0.016	0.020	0.024	0.031	0.042	0.064
	**0.20**	0.014	0.017	0.020	0.025	0.031	0.042	0.064
	**0.25**	0.015	0.018	0.021	0.025	0.032	0.043	0.064
	**0.30**	0.015	0.018	0.021	0.025	0.032	0.043	0.064
	**0.50**	0.016	0.018	0.022	0.026	0.032	0.043	0.065
**2000**	**0.05**	0.010	0.012	0.016	0.020	0.026	0.036	0.055
	**0.10**	0.011	0.013	0.016	0.020	0.026	0.036	0.055

	**0.15**	0.012	0.014	0.017	0.021	0.027	0.036	0.055
	**0.20**	0.012	0.014	0.017	0.021	0.027	0.037	0.055
	**0.25**	0.013	0.015	0.018	0.022	0.027	0.037	0.056
	**0.30**	0.013	0.015	0.018	0.022	0.028	0.037	0.056
	**0.50**	0.014	0.016	0.019	0.022	0.028	0.037	0.056
**2500**	**0.05**	0.009	0.011	0.014	0.018	0.023	0.032	0.049
	**0.10**	0.010	0.012	0.015	0.018	0.024	0.032	0.049
	**0.15**	0.010	0.012	0.015	0.019	0.024	0.033	0.050
	**0.20**	0.011	0.013	0.016	0.019	0.024	0.033	0.050
	**0.25**	0.011	0.013	0.016	0.019	0.024	0.033	0.050
	**0.30**	0.012	0.014	0.016	0.020	0.025	0.033	0.050
	**0.50**	0.012	0.014	0.017	0.020	0.025	0.033	0.050
**5000**	**0.05**	0.006	0.008	0.010	0.013	0.016	0.023	0.035
	**0.10**	0.007	0.008	0.010	0.013	0.017	0.023	0.035
	**0.15**	0.007	0.009	0.011	0.013	0.017	0.023	0.035
	**0.20**	0.008	0.009	0.011	0.013	0.017	0.023	0.035
	**0.25**	0.008	0.009	0.011	0.014	0.017	0.023	0.035
	**0.30**	0.008	0.010	0.011	0.014	0.017	0.023	0.035
	**0.50**	0.009	0.010	0.012	0.014	0.018	0.024	0.035

N: sample size. pi: assumed prevalence. P: probability of yes to the innocuous item.

SEs are identical for the ordinary CM (no subsamples) and for the two-subsample design with P and 1–P if the subsamples have equal size *N*/2.

SUPPLEMENTARY TABLE 7. Collaboration network in 10 years of empirical application of CM (2011 - 2021).

		**Description***	**Network statistics**
**Simple network parameters**	Number of nodes	Nodes demote unique authors in the dataset	108
	Number of edges	Edges denote the connections between any two authors in the network	278
	Number of components	Refers to the number of connected hubs, including pairs and single authors as unique component	18
	Network density	Network density shows how densely the network is populated, it ranges between zero and 1.	0.197
	Network diameter	The network diameter is the largest distance between two nodes.	5
	Network centralisation	Networks whose topologies resemble a star have a centralization value close to 1, whereas decentralized networks feature a centralization value close to zero.	0.325
	Network heterogeneity	The network heterogeneity reflects the tendency of a network contains hubs.	0.523
	Clustering coefficients	Network clustering coefficient is the average of the clustering coefficients for all nodes (authors) in the network. This measure is based on a network characteristic that all networks can be decomposed into triangles (3-way loops). The clustering coefficient of an author is the number of triangles (3-way loops) that involves any given author, relative to the maximum number of 3-way loops that could theoretically involve the same author. Clustering coefficient of one node (author) is always between zero (no connectivity) and 1 (maximum connectivity). Therefore, the high (close to 1) average clustering coefficient indicates high degree of connectivity, and vice versa.	0.862
**Complex network parameters/Node attributes**	Topological coefficient (average)	The topological coefficient is a relative measure for the extent to which an author shares neighbours with other author in the network (i.e., both authors co-authored a paper with a third author). Nodes that have one or no neighbours are assigned a topological coefficient of zero.	0.609
	Number of neighbours (average)	The connectivity of an author is defined by the number of its neighbours, which is the number of authors he/she is directly connected to via co-authored paper(s). Average number of neighbours indicates average connectivity of a node in network. In case an author has only one paper in	5.517
	Number of neighbours (range)		0–9.8

		the set, the number of neighbours is equivalent to the number of co-authors on that paper.	
	Stress centrality (average)	Stress centrality denotes the central points through which high number of the shortest patch travels through. High stress centrality values identify authors who play an important role in holding the collaborative network together.	22.94
	Stress centrality (range)		0–708
	Betweenness centrality (average)	The betweenness centrality of an author reflects the amount of control he/she exerts over the interactions of other authors in the network. Betweenness centrality favour authors connect hubs (subnetworks), rather than authors who are embedded inside a community.	0.039
	Betweenness centrality (range)		0–0.727
	Closeness centrality	Closeness centrality is a measure of how fast information spreads from one author to other reachable authors in the network.	0.680
	Eccentricity (average)	The maximum non-infinite length of a shortest path between an author and another author in the network. If an author is an isolated node, the eccentricity value is zero.	2.241
	Eccentricity (range)		0–5
	Number of directed/undirected edges (average)	This attribute counts the number of directed/undirected edges that are connected to an author.<br>Number of directed edges in this network is zero.	5.148
	Number of directed/undirected edges (range)		1–18
	Characteristics path length	Path length describes the *shortest path* between any two authors in the connected network.<br>The network diameter is the maximum length of shortest paths between two nodes. If a network is disconnected, its diameter is the maximum of all diameters of its connected components.<br>The network diameter and the shortest path length distribution is indicative of the small-world properties (a.k.a. 'six degrees of separation') of the network (Watts & Strogatz, 1998).	2.507

*Source and more detailed description: https://med.bioinf.mpi-inf.mpg.de/netanalyzer/help/2.7/index.html#complex

Watts, D. J., & Strogatz, S. H. (1998). Collective dynamics of 'small-world' networks. *Nature, 393*, 440–442.

SUPPLEMENTARY TABLE 8. Network node characteristics of the most influential authors

		Year of appearance		Node attributes										
**Author**	**1st author/all outputs**	**First**	**Last**	**1**	**2**	**3**	**4**	**5**	**6**	**7**	**8**	**9**	**10**	**11**
Chaman, R.	0/3	2015	2019	2.393	0.066	0.418	0.619	4	6.000	11	3	0.901	100	0.375
Haghdoost, A.	0/3	2016	2018	1.000	0.571	1.000	0.371	1	6.200	16	1	1.000	132	0.413
Hoffmann, A.	4/9	2015	2021	1.250	0.727	0.800	0.222	2	3.111	18	3	0.972	104	0.273
Jann, B.	1/5	2011	2018	1.643	0.574	0.609	0.303	3	6.250	14	2	0.954	708	0.240
Jerke, J.	2/3	2012	2021	2.214	0.254	0.452	0.467	4	5.667	9	3	0.913	296	0.315
Krumpal, I.	0/3	2011	2014	2.214	0.151	0.452	0.333	4	4.667	7	1	0.913	168	0.259
Mousavi, S.	0/4	2014	2019	2.393	0.066	0.418	0.619	4	6.000	13	3	0.901	100	0.375
Shamsipour, M.	1/3	2014	2019	1.714	0.419	0.583	0.407	3	7.000	17	3	0.949	468	0.318

1: Average shortest path length; 2: Betweenness centrality; 3: Closeness centrality; 4: Clustering coefficient; 5: Eccentricity; 6: Neighborhood connectivity; 7: Number of undirected edges; 8: Partner of multi-edged node pairs; 9: Radiality; 10: Stress; 11: Topological coefficient. For definitions, refer to Supplementary Table 7.
